# Adjunctive probiotic therapy sustains symptom relief in gastroesophageal reflux disease through gut microbiome-metabolome remodeling

**DOI:** 10.1128/msystems.01568-25

**Published:** 2026-01-29

**Authors:** Yingmeng Li, Qiong Li, Keyu Quan, Yong Xie, Ni Yang, Teng Ma, Longjin Zheng, Wei Zhou, Yalin Li, Hao Jin, Zhihong Sun, Yongfu Chen, Lai-Yu Kwok, Nonghua Lu, Weifeng Zhu, Wenjun Liu, Heping Zhang

**Affiliations:** 1 Inner Mongolia Key Laboratory of Dairy Biotechnology and Engineering, Inner Mongolia Agricultural University 117454 , Hohhot, Inner Mongolia, China; 2 Key Laboratory of Dairy Products Processing, Ministry of Agriculture and Rural Affairs, Inner Mongolia Agricultural University 117454 , Hohhot, Inner Mongolia, China; 3 Key Laboratory of Dairy Biotechnology and Engineering, Ministry of Education, Inner Mongolia Agricultural University 117454 , Hohhot, Inner Mongolia, China; 4 Collaborative Innovative Center of Ministry of Education for Lactic Acid Bacteria and Fermented Dairy Products, Inner Mongolia Agricultural University 117454 , Hohhot, Inner Mongolia, China; 5 State Key Laboratory for the Modernization of Classical and Famous Prescriptions of Chinese Medicine, Nanchang, China; 6 Research and Development Department, Jiangzhong Pharmaceutical Co., Ltd. 650127 , Nanchang, China; 7 Jiangxi University of Traditional Chinese Medicine 74582, Nanchang, China; 8 Department of Gastroenterology, The First Affiliated Hospital of Nanchang University 117970, Nanchang, China; Southern Medical University, Guangzhou, Guandong, China

**Keywords:** gastroesophageal reflux disease, proton pump inhibitor, rabeprazole, reflux disease questionnaire, multi-omics, microbiome-host interaction, short-chain fatty acids

## Abstract

Proton pump inhibitors (PPIs) are standard therapy for gastroesophageal reflux disease (GERD), but long-term use causes dysbiosis, gastrointestinal side effects, and symptom relapse after discontinuation. Probiotics may offer adjunctive benefits by modulating the gut ecosystem. The study aimed to evaluate the efficacy of a multi-strain probiotic (Lihuo) with rabeprazole in GERD and its impact on gut microbiota and metabolome. A randomized, double-blind, placebo-controlled trial was conducted in 120 GERD patients assigned to receive rabeprazole with either Lihuo (*n* = 64) or placebo (*n* = 56) for 8 weeks, followed by 4 weeks of probiotic or placebo alone. The primary outcome was change in the Reflux Disease Questionnaire (RDQ) score. Secondary outcomes included Gastrointestinal Symptom Rating Scale, endoscopic healing, and multi-omics profiling (shotgun metagenomics, phageome, and untargeted/targeted metabolomics). Compared with the placebo group, the probiotic group exhibited a pronounced 36.51% reduction in RDQ scores after 12 weeks of intervention (*P* = 0.017), alongside a higher numerical endoscopic healing rate (36.84% vs 12.50%; *P* = 0.365). Metagenomics revealed enrichment of *Bifidobacterium animalis*, *Lactiplantibacillus plantarum*, and *Clostridium* sp900540255, with reductions in *Bacteroides uniformis* and *Clostridium* Q *fessum*. Metabolomics showed increased γ-aminobutyric acid, succinate, citrulline, and short-chain fatty acids levels, with interesting microbe-metabolite correlations such as *Bifidobacterium animalis*-γ-aminobutyric acid and *Bacteroides fragilis*-succinate (*r* ≥ 0.30, *P* < 0.01). Our findings support that adjunctive probiotic therapy sustains post-PPI symptom relief, associated with targeted modulation of gut microbiota and bioactive metabolites.

**IMPORTANCE:**

Long-term proton pump inhibitor use in gastroesophageal reflux disease (GERD) may disrupt gut microbiota and cause symptom relapse after discontinuation. We found that adjunctive probiotic therapy sustained reflux reduction post-proton pump inhibitor. Probiotic use enriched beneficial taxa (*Bifidobacterium* and *Lactiplantibacillus plantarum*) and increased γ-aminobutyric acid, succinate, citrulline, and short-chain fatty acids. Strong correlations linked microbial shifts to metabolic and clinical improvements. This study demonstrates that adjunctive probiotic therapy enhances symptom control and supports microbial-metabolic homeostasis in GERD.

**CLINICAL TRIALS:**

This study is registered with the Chinese Clinial Trial Registry as ChiCTR2000038409.

## INTRODUCTION

Gastroesophageal reflux disease (GERD) is a common gastrointestinal disorder characterized by the pathological reflux of gastric contents into the esophagus, leading to chronic symptoms such as heartburn, regurgitation, retrosternal pain and, in more severe cases, mucosal damage (1). The global prevalence of GERD reaches up to 20% in Western populations, with an estimated 10.6% in China, imposing substantial clinical and economic burdens on healthcare systems (2–4). Proton pump inhibitors (PPIs) remain the first-line pharmacological therapy due to their potent acid-suppressive efficacy. However, a subset of patients continues to experience persistent or recurrent symptoms after discontinuing the PPI use, and long-term therapy is associated with gastrointestinal adverse effects such as diarrhea, abdominal pain, and constipation. Importantly, prolonged PPI administration has been shown to disrupt the composition and function of the gut microbiota, potentially exacerbating dysbiosis, compromising gut barrier integrity, and predisposing individuals to enteric infections and metabolic complications (5–7). Therefore, it is important to explore alternative or complementary strategies for GERD management.

Emerging evidence implicates the critical role of the gut microbiota in the pathophysiology of GERD (8). The bidirectional communication between the gut and the brain, mediated through the gut-brain axis, involves complex interactions among microbial metabolites, immune signaling, and neural pathways, all of which influence visceral sensitivity and symptom perception (9). Key microbial metabolites, such as γ-aminobutyric acid (GABA), serotonin (5-HT), short-chain fatty acids (SCFAs), and tryptophan derivatives, modulate intestinal barrier function, dampen inflammatory responses, and regulate gastrointestinal motility and immune homeostasis (10, 11). These findings suggest that targeting the gut microbiota and its functional output may represent a promising adjunctive strategy for GERD management.

Probiotics are defined as “live microorganisms that confer health benefits on the host when administered in adequate amounts” (12). The application of probiotics has gained recognition as a means to restore microbial balance and support gastrointestinal health. They have demonstrated efficacy in ameliorating symptoms across various functional and inflammatory gut disorders (6, 13). For example, *Lacticaseibacillus paracasei* F19 has been reported to prevent PPI-associated bowel symptoms (14). Notably, co-administration of *Bifidobacterium animalis* subsp. *lactis* MH-02 with PPIs led to faster symptom resolution, significant improvement in Gastrointestinal Symptom Rating Scale (GSRS) scores, and enhanced gut microbiota diversity in GERD patients (8). Despite these encouraging results, high-quality randomized controlled trials evaluating the clinical efficacy of specific probiotic strains in GERD, particularly those incorporating longitudinal microbiome and metabolomic analyses, are still limited.

The Lihuo probiotic formulation comprises three well-characterized strains: *Lacticaseibacillus paracasei* Zhang (Zhang), *Lactiplantibacillus plantarum* P9 (P9), and *Bifidobacterium animalis* subsp. *lactis* V9 (V9). These strains have individually and collectively demonstrated beneficial effects in gastrointestinal health. A combination of Zhang and V9 has been shown to alleviate constipation by modulating gut microbiota, reducing inflammation, and regulating metabolic pathways (15). The same formulation has also demonstrated efficacy in mitigating symptoms of ulcerative colitis through remodeling the intestinal mucosal microbiota (16). P9 supplementation has been associated with improved outcomes in chronic constipation, accompanied by increases in beneficial microbial taxa and metabolites (17). Furthermore, recent evidence indicates that P9 can ameliorate chronic diarrhea by reshaping the functional gut microbiome and metabolome (18). Given the ability of these strains to mitigate dysbiosis and restore microbial balance, this study hypothesized that adjunctive Lihuo probiotic therapy would enhance symptom relief and promote gut microbiota homeostasis in GERD patients.

Leveraging advanced multi-omics approaches, including shotgun metagenomics and untargeted metabolomics, this randomized, double-blind, placebo-controlled trial evaluates the clinical efficacy of Lihuo probiotic combined with rabeprazole and characterizes associated changes in the gut microbiota, phageome, and metabolome. This study aims to provide comprehensive evidence supporting the use of probiotics as a safe and effective adjunctive therapy in the management of GERD, bridging the gap between microbial modulation and clinically meaningful outcomes.

## MATERIALS AND METHODS

### Study design and participants

This randomized, placebo-controlled, double-blind, parallel-group clinical study was conducted at the First Affiliated Hospital of Nanchang University (Nanchang, China). The study protocol was approved by the Ethics Committee of the First Affiliated Hospital of Nanchang University (Approval No. IIT [2020] EC 003-2) and registered in the Chinese Clinical Trial Registry (No. ChiCTR2000038409). Written informed consent was obtained from all participants prior to enrollment in accordance with ethical guidelines.

Outpatients diagnosed with GERD were recruited and screened at the study site. Inclusion criteria were as follows: (i) male or female individuals aged 18–65 years and (ii) had gastroscopic examination performed at a local tertiary hospital within the previous three months, either confirmation of esophagitis (Los Angeles [LA] grades A, B, or C), or absence of esophagitis, accompanied by typical GERD symptoms, with a total score ≥ 12 on the Chinese version of Reflux Disease Questionnaire (RDQ) (19–21). Participants were excluded if they met any of the following criteria: (i) use of GERD-related medications, including acid inhibitors, antacids, prokinetics, gastric mucosal protectors, herbal remedies, or probiotics, within the past 2 weeks; (ii) elevated liver enzymes (aspartate aminotransferase or alanine aminotransferase > 2 × upper limit of normal), serum creatinine > 1 × upper limit of normal, heart failure, or electrocardiographic abnormalities; (iii) endoscopic diagnosis of peptic ulcer, gastrointestinal bleeding, esophageal varices, or upper gastrointestinal malignancy within the past three months; (iv) history of myocardial infarction, cerebral infarction, or malignant tumors; (v) prior gastroesophageal or duodenal surgery; (vi) active attempts to conceive, pregnancy, or breastfeeding; (vii) inability to cooperate with the study procedures, including inability to understand the informed consent form or refusal to provide personal information; (viii) known allergy to probiotics or any ingredient in the intervention products; and (ix) esophagitis secondary to gastric retention or pyloric obstruction.

### Randomization and blinding

Randomization was performed by two independent project administrators using a computer-generated random sequence created with the PROC PLAN procedure in SAS software (version 9.4; SAS Institute, Cary, NC, USA). Participants were assigned in a 1:1 ratio to either the probiotic or placebo group, and the allocation sequence was concealed from participants, treating physicians, and investigators throughout the study. Group assignments were sealed in opaque, sequentially numbered envelopes that remained unopened until after the primary data analysis was completed. To ensure distribution concealment, an independent project manager pre-labeled the probiotic and placebo sachets with unique identification numbers corresponding to the randomization sequence. The packaging and appearance of both products were identical, ensuring that all parties involved in participant care and outcome assessment remained blinded to group allocation.

### Study design and interventions

**Fig 1:**
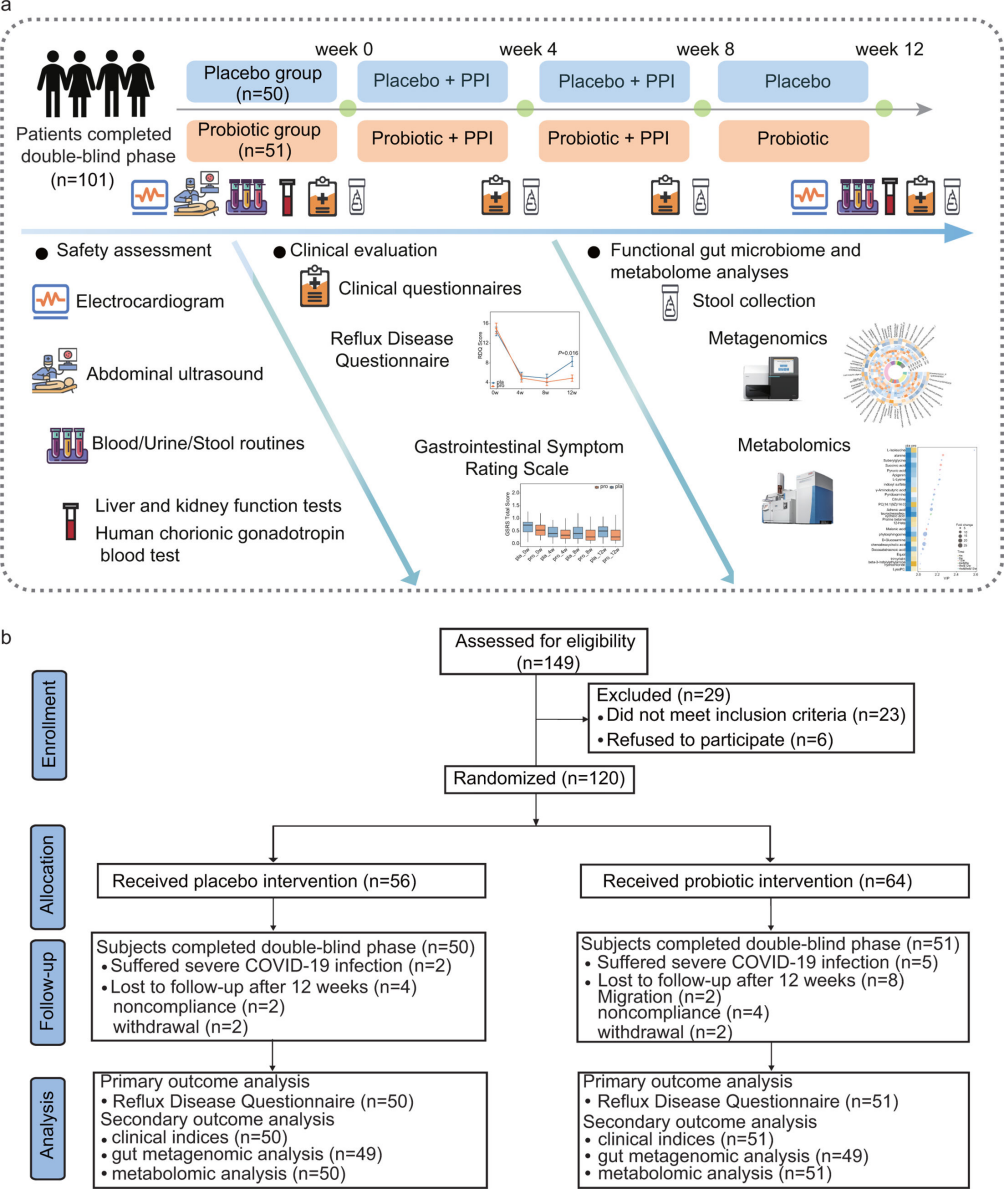
Study design and participant flow diagram. (**a**) Schematic representation of the three-phase study: a 2-week run-in period, an 8-week intervention phase with rabeprazole plus either probiotic or placebo, and a 4-week follow-up phase with continued probiotic or placebo after PPI discontinuation. (**b**) Consolidated Standards of Reporting Trials (CONSORT) flow diagram illustrating participant enrollment, allocation, follow-up, and final analysis in the intention-to-treat (ITT; *N* = 120) and per-protocol (PP; *N* = 101) populations. Exclusions were due to withdrawal, non-compliance, or migration. All analyses were performed under double-blind conditions.

The study consisted of three sequential phases: a 2-week run-in period, an 8-week intervention phase, and a 4-week follow-up phase after PPI discontinuation (Fig. 1a).

During the 8-week intervention phase, all participants received standard PPI therapy with rabeprazole (20 mg once daily before a meal; Eisai China Inc., Shanghai, China), in combination with either the Lihuo probiotic mixture or placebo. For the subsequent 4-week intervention phase, participants continued to receive only probiotic or placebo, with the PPI being discontinued.

The probiotic and placebo materials were provided by Jinhua Yinhe Biological Technology Co., Ltd. (Zhejiang, China). Each sachet of Lihuo probiotic powder contained a multi-strain formulation consisting of Zhang (8 × 10^9^ CFU), V9 (2.4 × 10^10^ CFU), and P9 (8 × 10^9^ CFU). The placebo powder was composed of maltodextrin and was matched to the probiotic material in color, texture, and taste. Participants in the probiotic group received two sachets of probiotic powder daily, administered after meals, whereas those in the placebo group received two sachets of placebo material. Throughout the study, participants were advised to maintain their usual lifestyle and dietary habits but were instructed to avoid alcohol, smoking, and stimulant foods such as spicy or fatty meals. Patients were also required to record the drug usage during each visit (weeks 4, 8, and 12), including the use of GERD-related medications (acid inhibitors, antacids, prokinetics, gastric mucosal protectors, and herbal remedies with the same efficacy as the above), or probiotics, prebiotics, and foods containing probiotics (like yogurt). All of these drugs and products were strictly prohibited. Treatment compliance was monitored by counting the number of returned empty packaging units (rabeprazole blister packs and sachets). Adherence was considered adequate if participants returned at least 80% of the distributed materials.

### Endpoint definitions

The primary outcome was the change in the total score of the Chinese version of the RDQ (22) assessed at baseline (week 0) and at weeks 4, 8, and 12. The RDQ is a self-reported instrument that evaluates four core symptoms, that is, heartburn, non-cardiac chest pain, acid regurgitation, and upwelling of stomach contents, over the preceding week (23). Each symptom is scored on two dimensions: frequency (0–5) and severity (0–5), where 0 indicates no symptoms and higher values reflect increasing frequency or severity. The total RDQ score was calculated as the sum of all eight item scores, with higher scores indicating greater symptom burden.

Secondary outcomes included changes in GSRS scores, body mass index (BMI), endoscopic healing status, and fecal metagenomic and metabolomic profiles. The GSRS comprised 15 items assessing gastrointestinal symptoms over the past week, grouped into five subscales: abdominal pain, reflux, diarrhea, indigestion, and constipation. Each item was rated from 0 (no discomfort) to 3 (severe discomfort). Subscale scores were calculated as the mean of the relevant items, and the total GSRS score was derived as the average of all 15 items. BMI was measured in the morning after an overnight fast, using standardized weight and height measurement instruments at each study visit. Gastroscopy was repeated at week 12 for participants who initially presented with reflux esophagitis. All procedures were performed by two senior clinicians following a standardized protocol, and esophagitis severity was classified according to the LA grading system (grades A–D) (24), with healing defined as resolution to LA grade 0 (no mucosal breaks) (25, 26).

Safety outcomes, including adverse events and results of laboratory tests, were recorded at each visit. Fecal samples were collected from all participants at weeks 0, 4, 8, and 12, immediately frozen at −80°C, and stored for subsequent analysis of the gut metagenome and metabolome.

### Fecal DNA extraction, shotgun metagenomic sequencing, and sequence quality control

Fecal DNA was extracted from stool samples using the Magnetic Soil and Stool DNA Kit (DP712; TIANGEN Biotech Co., Ltd., Beijing, China) following the manufacturer’s instructions. Extracted DNA was quantified and assessed for quality prior to library preparation. Shotgun metagenomic sequencing was performed on the Illumina Novaseq 6000 platform (Illumina Inc., San Diego, CA, USA) with paired-end 150 bp reads. Raw sequencing data were processed using Kneaddata (https://github.com/biobakery/kneaddata) to remove low-quality bases, adapter sequences, and host-derived reads. After quality filtering and host read depletion, an average of 10.43 Gbp of high-quality, non-host clean data was retained per sample.

### Bacterial genome assembly, binning, and functional prediction

Clean data were assembled into contigs using MEGAHIT (v1.2.9) with default parameters optimized for metagenomic data sets (27). Metagenome-assembled genomes (MAGs) were reconstructed through a multi-binner approach combining MetaBAT2 (28), VAMB (29), and DAS Tool (30), followed by refinement with custom scripts to minimize redundancy and improve bin completeness. The quality of the resulting MAGs was evaluated using CheckM (31), which were classified as high-quality (completeness ≥ 80%, contamination ≤ 5%), medium-quality (completeness ≥ 70%, contamination ≤ 5%), or partial-quality (lower thresholds). High-quality MAGs were dereplicated using dRep (32) with parameters -pa 0.95 and -sa 0.95 to cluster genomes at a 95% average nucleotide identity threshold and select the highest-quality representative genome from each cluster, generating species-level genome bins (SGBs). Taxonomic annotation of SGBs was accomplished using Kraken2 against the National Center for Biotechnology Information Non-Redundant Protein Sequence Database. The relative abundance of each SGB across samples was calculated using CoverM (https://github.com/wwood/CoverM; parameters: --min-read-percent-identity 0.95 --min-covered-fraction 0.4).

Gut metabolic modules (GMMs), conserved sets of enzymatic functions involved in key microbial metabolic processes, were inferred based on published literature and the MetaCyc database. Functional potential of the SGBs was assessed by annotating open reading frames (predicted using Prodigal) against the Kyoto Encyclopedia of Genes and Genomes (KEGG) database to identify metabolic modules. The distribution of these modules across SGBs was determined using Omixer-RPM (parameter: -c 0.66). Furthermore, the MelonnPan workflow was employed to the metagenomic data to predict the profile of bioactive microbial metabolites in the gut ecosystem, leveraging a curated gene-metabolite linkage database trained on metabolomic data sets.

### Phageome profiling and abundance analysis

Viral contigs were identified from assembled sequences exceeding 1,000 bp in length using VIBRANT (33) and CheckV (34). Only high-confidence viral contigs longer than 5,000 bp were retained for downstream analysis. These contigs were clustered into viral operational taxonomic units (vOTUs) using CD-HIT with thresholds of 95% nucleotide homology and 80% alignment coverage to account for intra-species variation (35). The resulting vOTUs were taxonomically annotated by comparison against the Metagenomic Gut Virus catalog (36), a comprehensive reference database containing 189,680 gut-derived viral genomes. The relative abundance of each vOTU was calculated using CoverM (https://github.com/wwood/CoverM) with the following parameters: --min-read-percent-identity 0.95, --min-read-aligned-percent 0.5, --proper-pairs-only, and –exclude-supplementary.

### Untargeted fecal metabolomics analysis by liquid chromatography-mass spectrometry

Fecal metabolite extraction was performed following protocols described in previous studies (18, 37). Briefly, fecal samples were homogenized in a methanol solution containing 2-chlorophenylalanine as an internal standard. The homogenates were subjected to ultrasonication for efficient metabolite extraction, followed by centrifugation at 12,000 rpm at 4°C for 10 min. The resulting supernatants were filtered through a 0.22 μm membrane for subsequent liquid chromatography-mass spectrometry analysis. Metabolomic profiling was performed using an Agilent 1290 Infinity LC ultra-high-performance liquid chromatography system (Agilent Technologies, Inc., Santa Clara, CA, USA) coupled to a Triple TOF 6600+ mass spectrometer (AB SCIEX, Framingham, MA, USA). Chromatographic separation was achieved on an ACQUITY UPLC BEH amide column (2.1 × 100 mm; Waters Corporation, Milford, MA, USA) maintained at 25°C. The mobile phase consisted of solvent A (25 mM ammonium acetate and 25 mM ammonia in water) and solvent B (acetonitrile) with the following gradient elution program: 0–0.5 min (95% B); 0.5–7 min (95%–65% B); 7–8 min (65%–40% B); 8–9 min (40% B); 9–9.1 min (40%–95% B); and 9.1–12 min (95% B). The flow rate was set at 0.5 mL/min, and the injection volume was 2 μL. The mass spectrometry was equipped with a heated electrospray ionization source and operated in both positive and negative ion modes. Full-scan mass spectra were acquired over the mass-to-charge (*m*/*z*) ranges of 60–1,000 and 25–1,000 *m*/*z*. Key instrument parameters included: ion source temperature of 600°C, curtain gas pressure of 30 psi, and ion spray voltage floating at 5,500 V for both ionization modes.

Raw mass spectrometry data were converted to the mzML format using ProteoWizard (version v3.0.8789) and processed using the R package XCMS for peak detection, alignment, and integration. Putative identification of significant metabolites was achieved by matching the Human Metabolome Database (HMDB; http://www.hmdb.ca/), LipidMaps (https://www.lipidmaps.org/), mzCloud (https://www.mzcloud.org/), Massbank (http://www.massbank.jp), and KEGG (https://www.kegg.jp/) databases (38). Pathway and functional enrichment analysis of identified metabolites was further conducted using the MetaboAnalyst platform (https://www.metaboanalyst.ca/MetaboAnalyst/home.xhtml).

### Targeted fecal metabolomics analyses for fecal SCFAs and neuroactive compounds

The concentrations of SCFAs were determined using gas chromatography-mass spectrometry (39). Briefly, stool samples were weighed and suspended in 1 mL of ultrapure water, vortexed for 10 s, and homogenized using a ball mill. The homogenates were subjected to ultrasonication on ice, followed by centrifugation at 10,000 × *g* for 15 min at 4°C. The supernatant was transferred to a fresh 2 mL microcentrifuge tube. Subsequently, 0.1 mL of 50% sulfuric acid and 0.8 mL of extracting solvent (methyl tert-butyl ether containing 2-methylvalerate at 25 mg/L as the internal standard) were added. The mixture was vortexed for 10 s and centrifuged again under the same conditions. The resulting organic phase (supernatant) was collected and transferred to a sample vial for subsequent analysis. Gas chromatography-mass spectrometry analysis was conducted using a Shimadzu GC2030-QP2020 NX system (Shimadzu Corporation, Kyoto, Japan) coupled with an Agilent J&W HP-FFAP capillary column (30 m × 250 μm × 0.25 μm; Agilent Technologies, Inc., Santa Clara, CA, USA). Helium was used as the carrier gas at a constant flow rate of 1.2 mL/min. The oven temperature program was as follows: initial temperature of 50°C (held for 1 min), increased to 150°C at 50°C/min (held for 1 min), then to 170°C at 10°C/min (0 min hold), 225°C at 25°C/min (1 min hold), and finally to 240°C at 40°C/min (1 min hold). The injector, transfer line, and ion source temperatures were set to 220°C, 240°C, and 240°C, respectively. Mass spectrometry was operated in electron impact ionization mode at −70 eV, with data acquired in scan and selected ion monitoring mode across an m/z range of 33–150.

Neuroactive compounds were analyzed using a validated targeted metabolomics method (40). Briefly, stool samples were weighed and mixed with 80 μL of pre-cooled extraction solvent (acetonitrile containing 0.1% formic acid at −20°C) and 20 μL of ultrapure water. The mixture was vortexed for 30 s, homogenized at 45 Hz for 4 min, and sonicated on ice for 5 min, repeated three times. After incubation at −20°C overnight, samples were centrifuged at 12,000 × *g* for 15 min at 4°C. A total of 80 μL of supernatant was mixed with 40 μL of 100 mM Na_2_CO_3_ solution and 40 μL of 2% benzoyl chloride in acetonitrile, followed by incubation at room temperature for 30 min to derivatize polar compounds. After adding 10 μL of internal standard, the samples were centrifuged again under the same conditions. A total of 40 μL of the resulting supernatant was mixed with 20 μL of deionized water and transferred to autosampler vials for ultra-high-performance liquid chromatography-tandem mass spectrometry analysis. Separation was performed on an ExionLC UHPLC system (AB SCIEX, Framingham, MA, USA) equipped with a Waters ACQUITY UPLC HSS T3 column (100 × 2.1 mm, 1.8 μm; Waters Corporation, Milford, MA, USA) maintained at 4°C. The mobile phase consisted of solvent A (water with 0.1% formic acid and 1 mM ammonium formate) and solvent B (acetonitrile). The autosampler was kept at 6°C, and the injection volume was 1 μL. Detection was carried out on a QTRAP 6500+ mass spectrometer (AB SCIEX, Framingham, MA, USA) in positive electrospray ionization mode, with ion spray voltage set to +5000 V, curtain gas at 35 psi, ion source temperature at 400°C, and nebulizer gases at 60 psi. Quantification was performed using multiple reaction monitoring.

### Statistical analyses

Sample size estimation was based on the primary outcome, the change in RDQ score, using effect size data from a previously published study (41) and input from clinical experts. An anticipated mean difference of 3.67 points in RDQ score between the probiotic and placebo groups was assumed, with a pooled standard deviation (SD) of 6.04. To achieve 80% power at a two-sided significance level of 5%, a minimum of 43 participants per group was required. After accounting for an estimated dropout rate of 20%, the target sample size increased to 53 participants per group.

All statistical analyses and data visualization were performed using R software (version 4.1.0), with figures refined in Adobe Illustrator. Efficacy was assessed in both the intention-to-treat (ITT) and per-protocol (PP) populations. The ITT population included all randomized participants, with missing symptom score data imputed using a multiple imputation procedure. The PP population excluded participants who discontinued the intervention or exhibited non-compliance (defined as <80% adherence to the assigned regimen). Baseline characteristics between the two treatment groups were compared using the χ^2^ test or Fisher’s exact test. Blood routine parameters, as well as liver and kidney function tests, were compared between groups using the two-sided Wilcoxon rank-sum test. Inter- and intra-group differences in RDQ scores, GSRS scores, BMI, SCFAs, neuroactive compounds, and other continuous variables were evaluated using the two-sided Mann-Whitney test (unpaired) or Wilcoxon rank-sum test (paired), respectively. A *P*-value < 0.05 was considered statistically significant.

For metagenomic and metabolomic analyses, Shannon and Simpson’s diversity indices, principal coordinates analysis (Bray-Curtis dissimilarity), principal component analysis, OPLS-DA, the adonis test, and Procrustes analysis were calculated and performed using several R packages, including ggplot2, ggpubr, vegan, optparse, and mixOmics. Inter- and intra-group differences in continuous variables were evaluated using the unpaired or two-sided paired Wilcoxon rank-sum test. Two-tailed Pearson correlation analysis was conducted to examine associations between clinical symptom scores, gut microbial taxa, and fecal metabolites.

## RESULTS

### Participant demographics and study cohort

A total of 149 individuals were assessed for study eligibility, of whom 29 were excluded, 23 due to failure to meet inclusion criteria and six due to withdrawal of consent prior to randomization. The remaining 120 participants provided written informed consent and were enrolled in the ITT population, with random assignment to either the probiotic group (*n* = 64) or the placebo group (*n* = 56). Over the 12-week trial period, 19 participants discontinued the intervention: 13 in the probiotic group and six in the placebo group, primarily due to COVID-19 infection, loss to follow up, or voluntary withdrawal. Consequently, 101 participants completed the trial per protocol, yielding a PP population of 51 in the probiotic group and 50 in the placebo group (Fig. 1b).

Baseline demographic and clinical characteristics for the ITT population (*N* = 120) are summarized in Table 1. Age and sex were self-reported and were not stratified during randomization. The mean (SD) age of patients in the probiotic and placebo groups was 46.33 (10.30) and 46.77 (9.66) years, respectively. The proportion of male subjects in the probiotic and placebo groups was 65.62% and 67.86%, whereas the proportion of female subjects was 34.38% and 32.14%, respectively. The mean (SD) body mass index values for the probiotic and placebo groups were 24.38 (3.19) and 23.93 (3.09), respectively. No statistically significant differences were observed between the probiotic and placebo groups in baseline characteristics, including age, BMI, sex distribution, history of allergies, smoking or alcohol use, comorbid medical conditions, or prior use of gastrointestinal medications (*P* > 0.05).

**TABLE 1:** Demographic and baseline characteristics of the intention-to-treat population^*a*^

Characteristics	Placebo group (*n* = 56)	Probiotic group (*n* = 64)	*P* value
Sex, no. (**%**)			
Male	38 (67.86)	42 (65.62)	0.80
Female	18 (32.14)	22 (34.38)	
Age, mean (SD), years	46.77 (9.66)	46.33 (10.30)	0.99
Smoking status, no. (%)			
Former smoker	2 (3.57)	1 (1.56)	0.46
Current smoker	18 (32.14)	17 (26.56)	
Never smoker	33 (58.93)	45 (70.31)	
Unknown	3 (5.36)	1 (1.56)	
Drinking status, no. (%)			
Yes	17 (30.36)	19 (29.69)	0.32
No	31 (55.36)	41 (64.06)	
Unknown	8 (14.28)	4 (6.25)	
Reflux Disease Questionnaire score, mean (SD)	15.59 (7.33)	15.75 (7.84)	0.99
Gastrointestinal Symptom Rating Scale score, mean (SD)			
Constipation	0.36 (0.36)	0.31 (0.37)	0.28
Diarrhea	0.36 (0.41)	0.35 (0.40)	0.98
Reflux	1.20 (0.76)	1.11 (0.64)	0.50
Abdominal pain	0.65 (0.55)	0.58 (0.51)	0.50
Indigestion	0.94 (0.57)	0.93 (0.62)	0.74
Total GSRS	0.69 (0.32)	0.65 (0.35)	0.36
Drug allergy, no. (%)			
Yes	1 (1.79)	7 (10.94)	0.07
No	55 (98.21)	57 (89.06)	
Other allergy, no. (%)			
Yes	1 (1.79)	1 (1.56)	>0.999
No	55 (98.21)	63 (98.44)	
Concomitant diseases, no. (%)			
Hypertension	6 (10.71)	7 (10.94)	0.97
Nephrolithiasis	1 (1.79)	2 (3.12)	>0.999
Thyroid nodule	1 (1.79)	3 (4.69)	0.62
Other diseases	15 (26.79)	16 (25.00)	0.82
Surgical history, no. (%)	21 (37.50)	26 (40.62)	0.73
Concomitant medication at baseline, no. (%)	13 (23.21)	14 (21.88)	0.86
Concomitant medication during trial, no. (%)	12 (21.43)	21 (32.81)	0.16

^*a*^ Categorical variables were compared using the Χ^2^ test or Fisher’s exact test, as appropriate, with no significant between-group differences observed.

### Probiotic supplementation sustains reflux relief after PPI cessation

**Fig 2:**
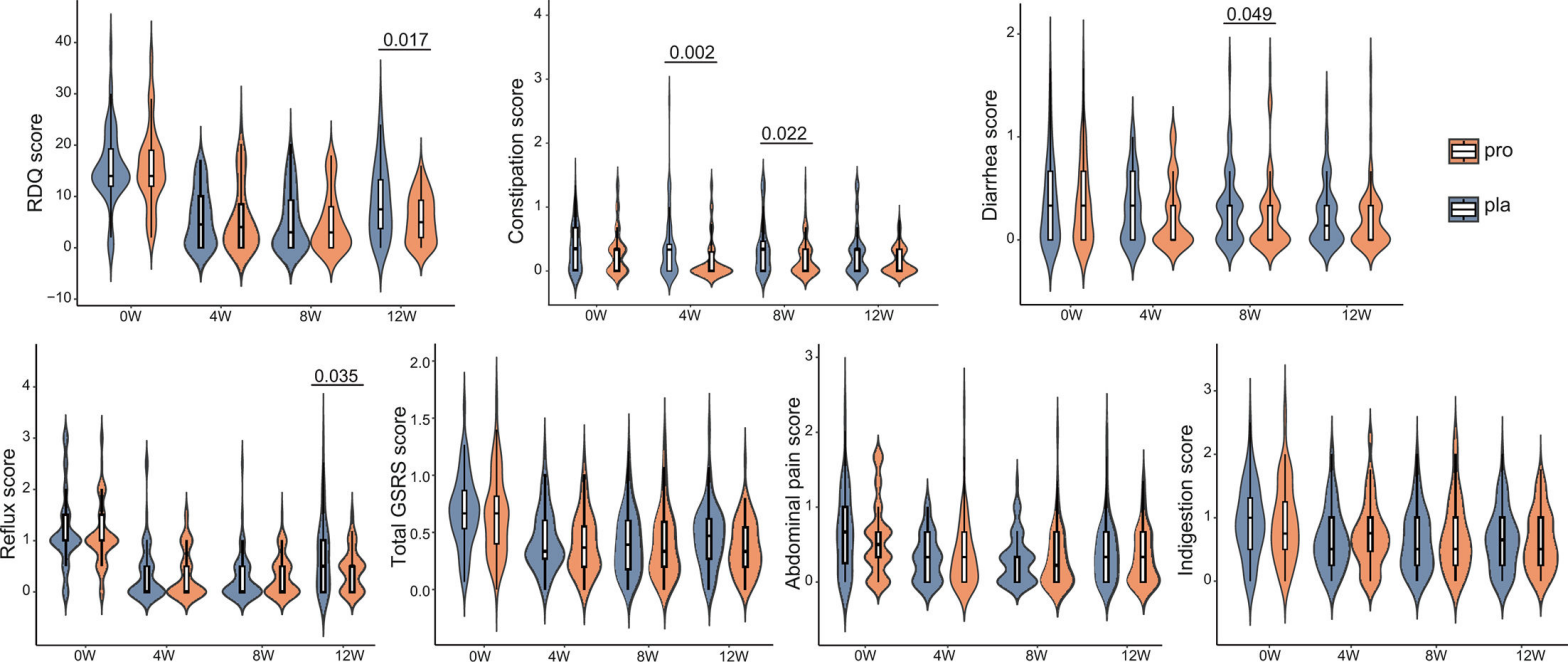
Probiotic supplementation sustains reflux relief after PPI cessation in the intention-to-treat population. Changes in RDQ score and GSRS total and subscores (constipation, diarrhea, reflux, abdominal pain, and indigestion) were evaluated over 12 weeks in the intention-to-treat population. Data are shown for the probiotic (pro; *n* = 64) and placebo (pla; *n* = 56) groups at weeks 0 (0w), 4 (4w), 8 (8w), and 12 (12w). Intergroup differences at each time point were assessed using two-sided Wilcoxon rank-sum tests, with significant *P*-values (*P* < 0.05) indicated. In the violin plots, the box represents the interquartile range, with the median indicated by the internal line. Whiskers extend to the lowest and highest values within 1.5 times the interquartile range.

At baseline (week 0), no significant differences were observed between the probiotic and placebo groups in RDQ and GSRS scores (*P* > 0.05, Fig. 2; Fig. S1; Tables S1 and S2). During the 8-week phase of combined probiotic and rabeprazole therapy, ITT analysis revealed that RDQ and GSRS scores significantly decreased in both probiotic and placebo groups (*P* < 0.05), although no significant inter-group differences were observed in RDQ score at week 8 (4.92 ± 5.70 vs. 5.20 ± 5.87, *P* > 0.05), the probiotic group experienced significantly greater improvements in gastrointestinal symptoms compared with the placebo group (Fig. 2; Table S1), with a significant reduction in constipation scores at weeks 4 and 8 by 54.05% and 43.24%, respectively, and a significant 22.58% decrease in diarrhea scores at week 8. Per-protocol analysis confirmed these trends, with statistically significant reductions in both constipation scores at weeks 4 and 8 and diarrhea scores at week 8 (*P* < 0.05; Fig. S1, Table S2).

During the subsequent 4-week follow-up phase, after discontinuation of rabeprazole, patients in the probiotic group continued to show superior symptom outcomes. At week 12, no significant difference was observed in RDQ score of the probiotic group compared with week 8, whereas the placebo group showed significant increase compared with week 8 (*P* < 0.05), and the probiotic group demonstrated a 36.51% greater reduction in RDQ score compared with the placebo group at week 12 (5.67 ± 4.59 vs. 8.93 ± 7.10; *P* = 0.017; Fig. 2), with the PP analysis revealing an even more pronounced 44.98% relative improvement (*P* = 0.007; Fig. S1). Additionally, the probiotic group also exhibited a 41.89% reduction in reflux scores compared with the placebo group at week 12 (*P* = 0.035; Fig. 2; Table S1). In the PP analysis, this improvement was accompanied by significant reductions in both reflux and total GSRS scores (*P* < 0.05; Fig. S1, Table S2), indicating sustained symptomatic benefit. These findings suggest that continued probiotic supplementation after PPI withdrawal may support prolonged symptom relief and prevent early relapse. No significant differences were noted between groups in abdominal pain, indigestion, or BMI throughout the trial in either ITT or PP analyses (*P* > 0.05; Fig. 2; Fig. S1), indicating a selective effect of the probiotic formulation on bowel motility-related and reflux-specific symptoms.

Endoscopic evaluations were available for 27 participants at week 12 (Fig. S2). In the probiotic group, the proportion of patients with LA grade A esophagitis decreased from 73.68% to 36.84%, and those with grade B declined from 21.05% to 15.79%. In contrast, in the placebo group, grade A decreased from 62.50% to 50.00%, and grade B remained unchanged. One patient in each group had LA grade C, with no cases of grade D observed. Although the endoscopic healing rate (resolution to LA grade 0) was higher in the probiotic group (36.84%) than in the placebo group (12.50%), the difference did not reach statistical significance (*P* = 0.365). Nevertheless, these results suggest a potential role for probiotics in supporting mucosal recovery, particularly in mild-to-moderate reflux esophagitis.

### Probiotic supplementation is safe and well-tolerated in GERD patients

Comprehensive safety assessments were conducted before and after the intervention, including blood, urine, and stool routine tests, liver and kidney function evaluations, and electrocardiograms. No clinically significant abnormalities were observed in either the probiotic or placebo group at baseline or post-intervention (Table S3). A total of 25 non-severe adverse events were recorded during the study period (Table S4). Reported events included upper respiratory tract infections, gingivitis, diarrhea, dizziness, sore throat, and bloating. The overall incidence of adverse effects was non-significantly higher (*P* = 0.10) in the probiotic group (17 events; 26.56%) compared with the placebo group (eight events; 14.29%). One serious adverse event, vocal cord granuloma, occurred in a participant assigned to the placebo group. The event required hospitalization but resolved completely with standard medical management. None of the adverse events were considered related to the probiotic formulation or the trial procedures after independent clinical evaluation. These findings demonstrate that adjunctive supplementation with the Lihuo probiotic mixture is safe, does not adversely affect systemic or gastrointestinal parameters, and exhibits a safety level comparable to that of a placebo in patients with GERD.

### Probiotic coadministration remodels the gut microbiota in GERD patients

**Fig 3:**
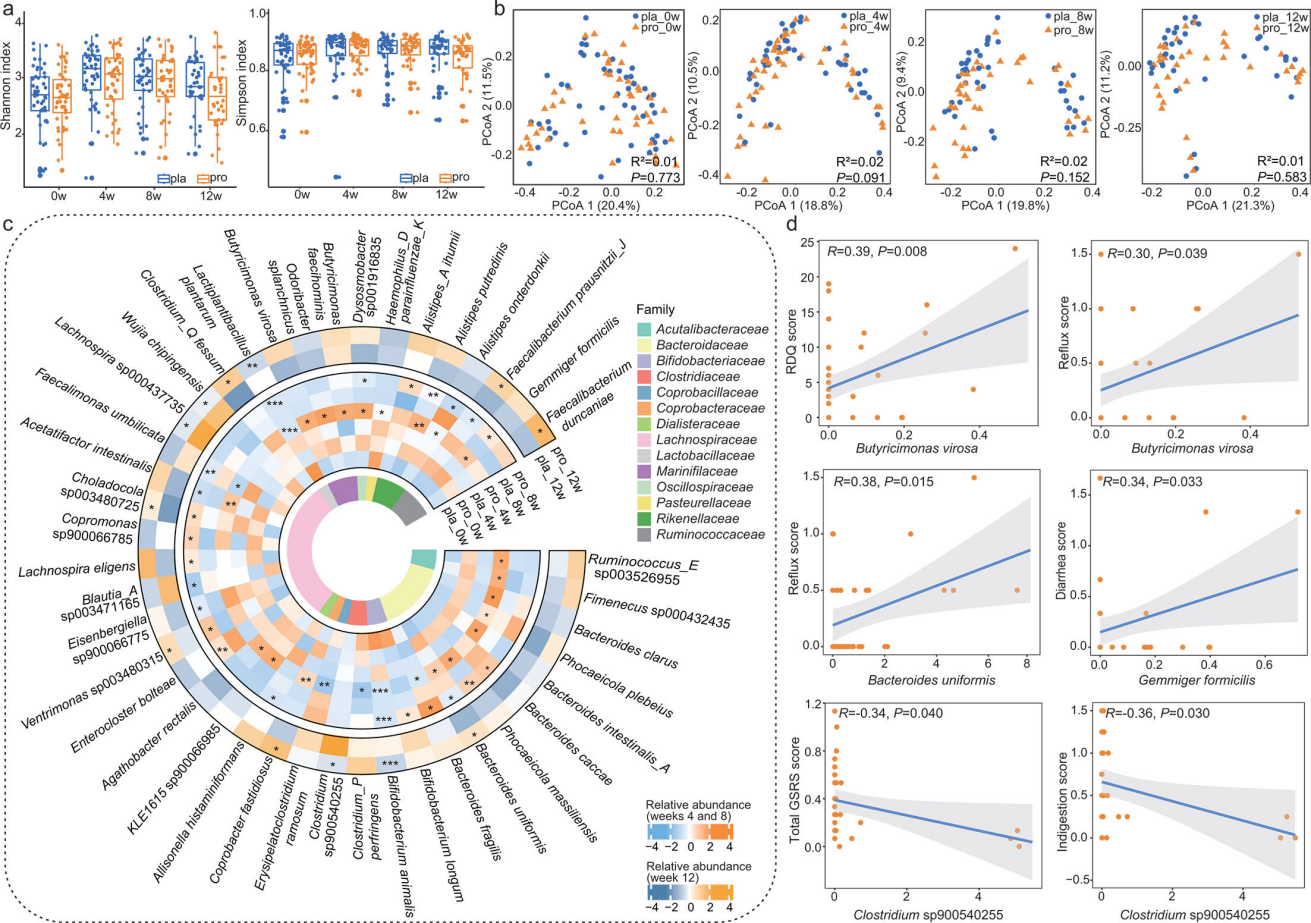
Probiotic supplementation reshapes the gut microbiome with functional correlations to symptom relief. (**a**) Boxplots showing alpha diversity (Shannon and Simpson’s diversity indices) in the probiotic (pro; *n* = 49) and placebo (pla; *n* = 49) groups at weeks 0 (0w), 4 (4w), 8 (8w), and 12 (12w). (**b**) Principal coordinates analysis (Bray-Curtis dissimilarity) of the gut microbiota, with Adonis test results shown in the lower right corner of each plot. (**c**) Circular plot illustrating the relative abundance of significantly differential species-level genome bins (SGBs) between groups during the intervention (pro, *n* = 49; pla, *n* = 49). The color scales represent relative abundance, ranging from high (red) to low (blue). Statistical differences were assessed using two-sided Wilcoxon rank-sum tests (* *P* < 0.05; ** *P* < 0.01; *** *P* < 0.001). (**d**) Pearson correlation scatter plots showing associations between differentially abundant SGBs and clinical symptom scores in the probiotic group at weeks 4, 8, and 12.

No significant differences in alpha diversity, assessed via Shannon and Simpson’s diversity indices, were observed between the probiotic and placebo groups at any time point (*P* > 0.05; Fig. 3a). Similarly, the principal coordinates analysis (Bray-Curtis dissimilarity) did not reveal distinct clustering of microbial communities between groups across the intervention period (Fig. 3b), indicating no large-scale structural shifts in overall microbiota composition attributable to probiotic supplementation.

Despite the absence of global community shifts, detailed analysis revealed 42 differentially abundant SGBs with an average relative abundance ≥ 0.01% over the intervention period (Table S5). No significant baseline imbalances in these SGBs were detected between groups, confirming pre-intervention comparability. In the 8-week phase of combined probiotic and rabeprazole therapy, 37 SGBs exhibited significant enrichment in the probiotic group compared with placebo, including *Bifidobacterium animalis, Faecalimonas umbilicata,* and *Lactiplantibacillus plantarum* (*P* ≤ 0.05 for all comparisons; Fig. 3c), all of which are associated with beneficial metabolic activities, including SCFA production and gut barrier support. Conversely, taxa such as *Gemmiger formicilis* and *Bacteroides uniformis*, previously linked to dysbiosis and pro-inflammatory potential, were significantly reduced in abundance (*P* ≤ 0.05 for all comparisons; Fig. 3c). Following the 4-week probiotic-only phase (week 12), several taxonomic shifts persisted. *Bifidobacterium animalis*, *Lactiplantibacillus plantarum*, and *Clostridium* sp900540255 were enriched in the probiotic group, whereas *Bacteroides uniformis* and *Clostridium* Q *fessum* remained lower compared with placebo (*P* ≤ 0.05 for all comparisons; Fig. 3c). These findings suggest that probiotic supplementation induces selective, persistent remodeling of key functional taxa, even after discontinuation of PPI therapy.

To explore potential links between microbial dynamics and clinical outcomes, Pearson correlation analysis was performed between SGB abundances and symptom scores (Fig. 3d). At week 4, *Butyricimonas virosa* showed significant positive correlations with both RDQ score (*r* = 0.39, *P* = 0.008) and reflux score (*r* = 0.30, *P* = 0.039). At week 8, *Bacteroides uniformis* correlated positively with reflux severity (*r* = 0.38, *P* = 0.015), and *Gemmiger formicilis* was positively associated with diarrhea score (*r* = 0.34, *P* = 0.033). By week 12, *Clostridium* sp900540255 exhibited significant negative correlations with total GSRS score (*r* = −0.34, *P* = 0.040) and indigestion score (*r* = −0.36, *P* = 0.030), suggesting a protective role in gastrointestinal symptom control. Collectively, these results demonstrate that adjunctive probiotic therapy drives targeted enrichment of beneficial taxa and suppression of potentially detrimental ones. The observed correlations between specific SGBs and symptom improvement support a functional link between microbiota modulation and clinical outcomes in GERD patients.

### Probiotic coadministration modulates the gut phageome in GERD patients

A comprehensive phageome analysis identified 42,554 non-redundant vOTUs from fecal samples. Of these, 33.79% were taxonomically classified into 12 known bacteriophage families. Genome quality assessment revealed 640 (4.45%) complete, 2,289 (15.92%) high-quality, and 11,451 (79.63%) medium-quality viral genomes (Fig. S3a). Consistent with findings in the bacterial microbiota, no significant differences in alpha diversity (Shannon and Simpson’s diversity indices) or beta diversity, assessed via principal coordinates analysis, were observed in the overall phageome community between the probiotic and placebo groups at any time point (*P* > 0.05; Fig. S3b and c). Moreover, a strong correlation was observed between the Shannon diversity of the bacterial microbiota and the phageome (*R* = 0.888, *P* < 0.001; Fig. S3d), indicating coordinated dynamics between bacterial hosts and their associated phages. This relationship was further validated by Procrustes analysis, which revealed a high degree of congruence in compositional variation between bacterial and viral communities (*R* = 0.939, *P* = 0.001; Fig. S3e), supporting a tightly coupled, cooperative interaction within the gut ecosystem.

The overall phage community structure remained stable across interventions, comprising ten different families, with *Siphoviridae* and *Myoviridae* dominating across all subgroups, collectively representing the core gut phageome (Fig. S3f). Notably, although overall phage profiles were highly similar between groups, differential abundance analysis revealed significant changes in specific vOTUs following probiotic supplementation (*P* < 0.05 in all cases; Fig. S3g). These findings indicate that adjunctive probiotic therapy does not broadly reorganize the gut virome but can selectively influence specific bacteriophage populations.

### Probiotic coadministration modulates GMM and predicted bioactive metabolite profiles

A total of 76 GMMs were discerned, and 14 GMMs exhibited significant differential abundance between the probiotic and placebo groups over the intervention period (*P* < 0.05; Fig. S4a). After 4 weeks of combined probiotic and rabeprazole therapy, the probiotic group showed significantly higher abundances of microbial pathways involved in tryptophan synthesis, histamine degradation, vitamin K_2_ synthesis II, and acetate synthesis I compared with the placebo group. Conversely, pathways related to GABA degradation and vitamin B_12_ biosynthesis I were significantly reduced in the probiotic group (*P* < 0.05 in all cases; Fig. S4a). By week 8, the probiotic group demonstrated further enrichment in pathways associated with the synthesis of acetate, isovaleric acid, and vitamins K_2_, B_6_, and B_12_ (*P* < 0.05 in all cases; Fig. S4a). At week 12, the leucine degradation pathway remained significantly enriched in the probiotic group (*P* = 0.017; Fig. S4a), suggesting selective modulation of neuroactive, SCFAs, and amino acid-related microbial metabolic potential.

To translate metagenomic profiles into biologically interpretable metabolite predictions, the MelonnPan workflow was applied. Based on the taxonomic composition of the gut microbiota, MelonnPan predicted 80 metabolites across all samples. Although non-metric multidimensional scaling revealed no significant global separation in predicted metabolite profiles between groups (Fig. S4b), targeted examination identified eight metabolites with significant intergroup differences during the intervention (Table S6). Notably, the probiotic group exhibited elevated levels of chenodeoxycholate and phytosphingosine at week 4 (*P* = 0.019 and 0.046, respectively; Fig. S4c), followed by increased glutamate and citrulline at week 8 (*P* = 0.044 and 0.020, respectively; Fig. S4c). Phytosphingosine has anti-inflammatory and antimicrobial properties, and chenodeoxycholate, a primary bile acid, may influence gut motility and microbiota composition. Glutamate is a precursor for GABA synthesis and plays a role in intestinal barrier function, whereas citrulline is linked to nitric oxide production and gastrointestinal motility. These results demonstrate that probiotic supplementation directly modulates the functional potential of the gut microbiome, altering both metabolic pathways and the abundance of predicted bioactive metabolites.

### Probiotic coadministration reshapes the fecal metabolome and reveals microbe-metabolite interactions in GERD patients

**Fig 4:**
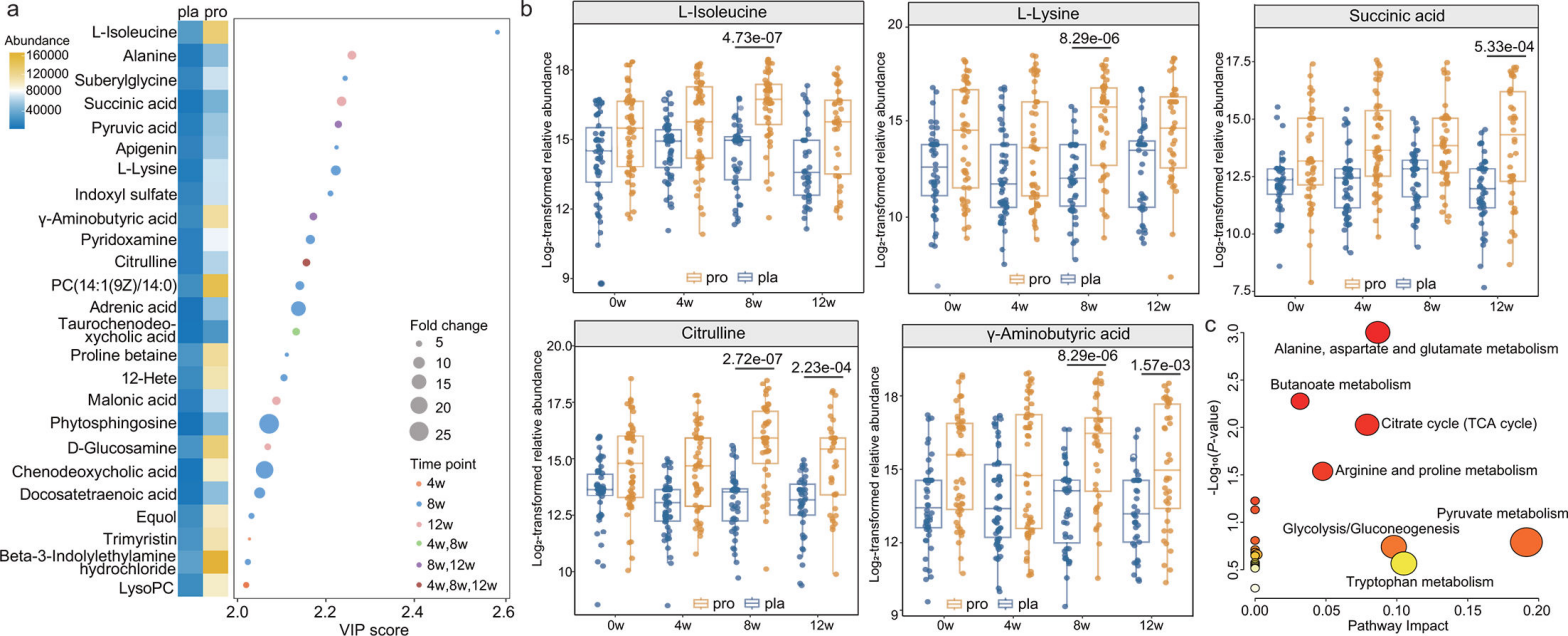
Probiotic supplementation reshapes the fecal metabolome and enriches key metabolic pathways. (**a**) Heatmap showing differentially abundant fecal metabolites (variable importance in projection score > 2.0, *P* < 0.05, and fold change > 4 or < 0.25) between the probiotic (pro; *n* = 51) and placebo (pla; *n* = 50) groups at weeks 4 (4w), 8 (8w), and/or 12 (12w). Circle size reflects the magnitude of fold change, whereas color indicates the time point at which significant intergroup differences were observed. The color scale represents metabolite abundance level. (**b**) Boxplots of relative abundance of significant probiotic-responsive metabolites, including L-isoleucine, L-lysine, succinic acid, citrulline, and γ-aminobutyric acid. The box represents the interquartile range, with the median indicated by the internal line. Whiskers extend to the lowest and highest values within 1.5 times the interquartile range. Statistical differences between groups were evaluated using two-sided Wilcoxon rank-sum tests, with Benjamini-Hochberg correction. (**c**) Pathway enrichment analysis based on the differentially abundant fecal metabolites. Circle size represents the enrichment ratio (larger circles = greater enrichment); color reflects the -log_10_ (*P*-value), with red indicating higher statistical significance and yellow indicating lower significance.

Next, changes in the fecal metabolome during the intervention were analyzed. Instrument performance and analytical stability were confirmed by principal component analysis of quality control samples, which clustered tightly in the score plot (Fig. S5a), indicating minimal system drift and high reproducibility of the untargeted metabolomics workflow. The OPLS-DA effectively differentiated the metabolic profiles of the probiotic and the placebo groups across multiple time points (Fig. S5b). Metabolite-level differential analysis was conducted using a stringent threshold: variable importance in projection score > 2.0, *P* < 0.05, and fold change > 4 or < 0.25. This approach identified 25 significantly differential fecal metabolites between groups, with none of them exhibiting significant baseline differences (Fig. 4a; Table S7). At week 4, the probiotic group exhibited significantly higher levels of trimyristin (a triglyceride with potential anti-inflammatory properties) and taurochenodeoxycholic acid (a taurine-conjugated bile acid involved in lipid digestion and gut signaling) compared with the placebo group (*P* < 0.05; Fig. 4a). By week 8, several amino acids, including L-isoleucine and L-lysine, were significantly enriched in the probiotic group (*P* < 0.05; Fig. 4a and b), reflecting enhanced microbial amino acid synthesis or reduced host utilization. At the end of the 12-week intervention, succinic acid, an intermediate of the tricarboxylic acid cycle and a substrate for gluconeogenesis and butyrate production, was significantly elevated in the probiotic group (*P* < 0.05; Fig. 4a and b). Notably, citrulline and GABA were consistently enriched during both the combined probiotic-PPI phase and the subsequent probiotic-only phase (*P* < 0.05; Fig. 4b), suggesting sustained microbial metabolic activity even after acid suppression was discontinued. Citrulline is a key intermediate in the urea and nitric oxide synthesis pathways, linked to intestinal barrier integrity and motility, whereas GABA is a neuroactive metabolite with anti-inflammatory and gut-brain axis modulatory functions. Pathway analysis of the 25 differential metabolites revealed significant enrichment in several key metabolic networks. The most impacted pathways included alanine, aspartate, and glutamate metabolism, arginine and proline metabolism, butanoate metabolism, pyruvate metabolism, glycolysis/gluconeogenesis, and the citrate cycle (TCA cycle) (Fig. 4c). These findings indicate that probiotic supplementation drives functional shifts in microbial metabolism related to energy production, amino acid homeostasis, and SCFA biosynthesis.

To explore the potential microbial drivers of these metabolic changes, two-tailed Pearson correlation analysis was conducted between significantly altered metabolites and bacterial species (Fig. S5c). Citrulline showed significant positive correlations with *Bifidobacterium animalis* (*r* = 0.30, *P* = 0.002) and *Bifidobacterium longum* (*r* = 0.40, *P* < 0.001), whereas GABA correlated positively with *Bifidobacterium animalis* (*r* = 0.30, *P* = 0.005) and *Faecalimonas umbilicata* (*r* = 0.40, *P* < 0.001). Moreover, succinic acid demonstrated a significant positive correlation with *Bacteroides fragilis* (*r* = 0.33, *P* < 0.001). These correlations suggest a cooperative relationship between specific probiotic-associated taxa and bioactive metabolite production. Collectively, these results demonstrate that adjunctive probiotic therapy induces significant and sustained remodeling of the fecal metabolome in GERD patients, with notable increases in metabolites linked to gut health, neuroregulation, and energy metabolism.

### Probiotic coadministration modulates fecal levels of neuroactive compounds and short-chain fatty acids

**Fig 5:**
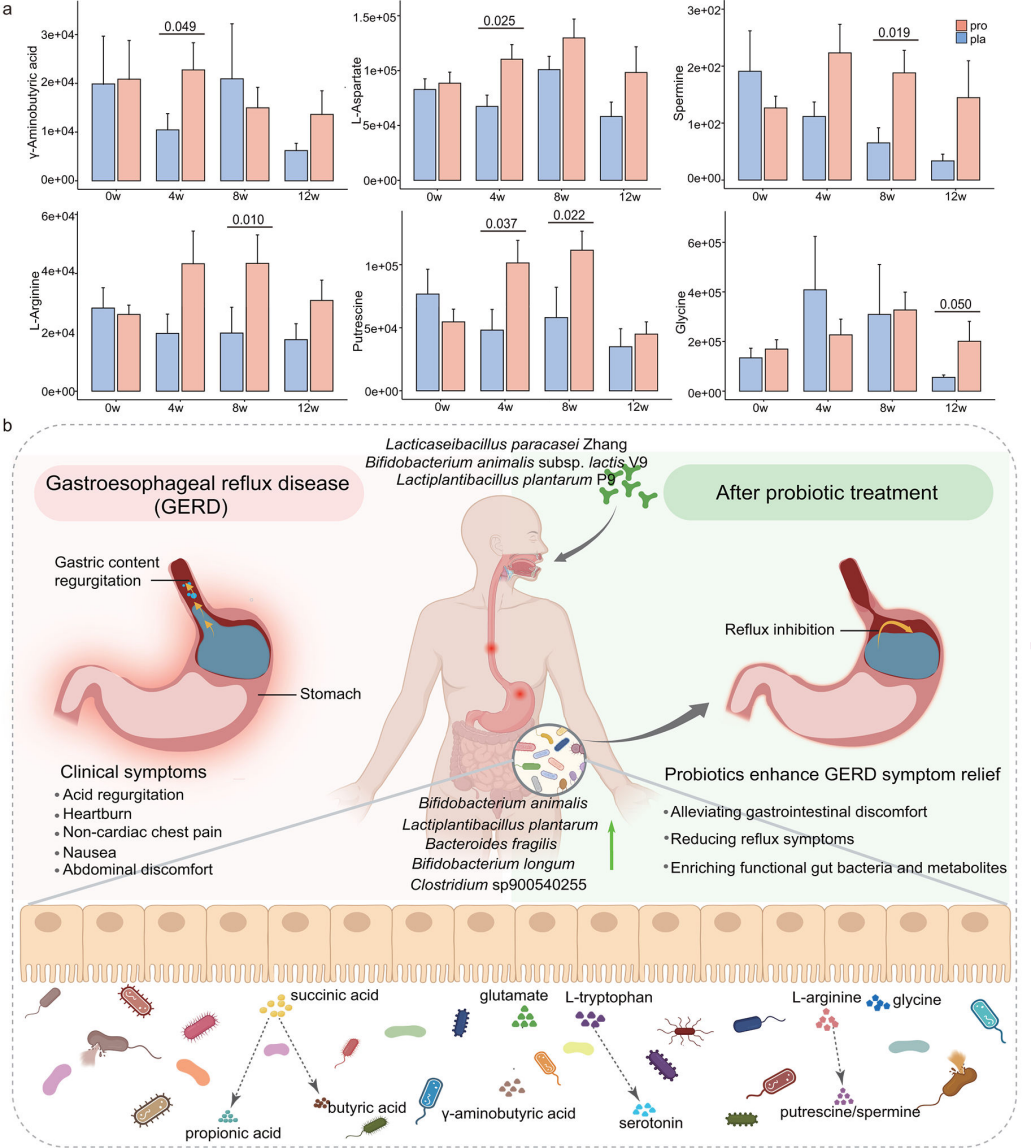
Integrated multi-omics framework links probiotic coadministration to symptom improvement in GERD. (**a**) Bar charts showing the levels of key fecal metabolites (in arbitrary units) in the probiotic (pro; *n* = 51) and placebo (pla; *n* = 50) groups at weeks 0 (0w), 4 (4w), 8 (8w), and 12 (12w). Error bars represent the standard error of the mean. Statistical significance was evaluated using two-sided Wilcoxon rank-sum tests, with *P-*values displayed for comparisons reaching significance (*P* < 0.05). (**b**) Conceptual model summarizing the effects of Lihuo probiotic supplementation. Probiotics alleviate GERD symptoms, modulate gut microbial composition, and sustain enrichment of beneficial taxa and metabolites, such as γ-aminobutyric acid, serotonin, and short-chain fatty acids, supporting continued reflux symptom relief. This integrative framework illustrates the functional link between microbial modulation and clinical outcomes.

Targeted metabolomics analysis revealed that probiotic supplementation significantly altered fecal concentrations of key microbial metabolites associated with gut-brain axis signaling and energy metabolism (Fig. 5A; Table S8). Compared with the placebo group, the probiotic group exhibited significantly elevated levels of GABA and L-aspartate at week 4 and increased concentrations of spermine and L-arginine at week 8. Notably, putrescine levels were significantly higher in the probiotic group at both weeks 4 and 8 (*P* < 0.05), whereas glycine levels showed a trend toward increase at week 12 (*P* = 0.050), following the probiotic-only phase.

Longitudinal within-group analysis further revealed dynamic metabolic shifts in the probiotic group over the 12-week intervention (Table S8). By week 8, significant increases were observed in fecal levels of butyric acid, propionic acid, L-leucine, L-valine, putrescine, and methionine compared with baseline (week 0; *P* < 0.05). L-tryptophan, a precursor for 5-HT and microbial indole derivatives, was significantly higher at week 8 than at week 4, and butyric acid remained elevated at week 12 relative to baseline (*P* < 0.05), indicating sustained enhancement of beneficial microbial metabolic activity. In contrast, 5-HT levels were significantly reduced at weeks 8 and 12 compared with baseline (*P* < 0.05), implicating a potential shift in host serotonergic signaling. Additionally, several metabolites, including L-glutamine, L-tryptophan, putrescine, and methionine, decreased at week 12 compared with week 8 (*P* < 0.05), which may reflect feedback regulation, substrate depletion, or microbial community adaptation following PPI discontinuation. Collectively, these results highlight the functional impact of probiotic intervention on the gut metabolome, linking microbial activity to host-relevant metabolic pathways that may underlie the observed clinical benefits in GERD patients.

## DISCUSSION

Over the past decades, GERD has emerged as a prevalent gastrointestinal disorder worldwide (2). PPIs remain the cornerstone of GERD management due to their potent acid-suppressive effects. However, long-term PPI use is associated with gastrointestinal side effects, increased susceptibility to intestinal infections, and high relapse rates upon discontinuation (42, 43). In this randomized, double-blind, placebo-controlled trial, the efficacy of adjunctive probiotic therapy, using a multi-strain formulation (Lihuo), in combination with rabeprazole for the treatment of GERD was evaluated.

An intriguing finding of this study is that probiotic coadministration significantly supports sustained symptom relief after PPI withdrawal, concomitant with favorable shifts in gut microbial composition and metabolic profile (Fig. 5b). Importantly, during the 4-week probiotic-only phase, after rabeprazole discontinuation, the RDQ score decreased by 35.21% (ITT) and 40.87% (PP) in the probiotic group compared with placebo, indicating that continued probiotic use may help prevent symptom rebound. In addition, the probiotic group also exhibited marked improvements in both reflux and total GSRS scores. These outcomes are consistent with prior reports showing that supplementation with *Bifidobacterium bifidum* YIT 10347 for 4 weeks improves postprandial discomfort (44) and that daily intake of heat-killed *Lactobacillus johnsonii* No. 1088 alleviates heartburn symptoms (45). Moreover, our multi-omics data provide a strong, direct biological rationale for the sustained reflux relief. The significant enrichment of beneficial taxa (such as *Bifidobacterium animalis*) and their strong correlations with bioactive metabolites outline a plausible pathway for reducing visceral hypersensitivity and strengthening mucosal integrity independent of bowel habit changes. For instance, the correlation between *Bifidobacterium animalis* and increased GABA (*r* = 0.30, *P* = 0.005) is critical, as GABA is a key inhibitory neurotransmitter known to modulate esophageal sensitivity (46). This is consistent with the notable numerical difference in endoscopic healing rates (36.84% vs. 12.50%, *P* = 0.365) in favor of the probiotic group, which suggests that the intervention may be promoting mucosal repair. This effect on the esophageal tissue itself is likely a direct result of the systemic anti-inflammatory and barrier-strengthening effects of the modulated microbiome and its metabolites (e.g., SCFAs, succinate). Collectively, these data highlight the therapeutic potential of integrating probiotics with standard PPI therapy to enhance symptom control and improve patient outcomes in GERD through distinct yet complementary mechanisms.

Probiotics exert their health benefits largely through modulating the gut microbiota and its functional output (47). In this study, microbial metagenomic analysis revealed that probiotic supplementation led to significant enrichment of beneficial bacterial taxa, including *Bifidobacterium animalis*, *Lactiplantibacillus plantarum*, *Bacteroides fragilis*, *Bifidobacterium longum*, and *Clostridium* sp900540255, along with specific vOTUs associated with the *Siphoviridae* and *Myoviridae* families. Notably, *Bifidobacterium animalis* subsp. *lactis* MH-02 has been shown to reduce GSRS scores and extend remission in patients with reflux esophagitis (8). Many of the enriched taxa, including *Lactiplantibacillus plantarum, Bacteroides fragilis*, *Bifidobacterium longum*, and *Clostridium* sp900540255, are known producers of SCFAs, particularly butyrate and acetate, which play critical roles in maintaining gut homeostasis, reinforcing epithelial barrier integrity, and suppressing pathogenic microbes (8, 13, 48–50). Furthermore, our findings indicate that probiotic treatment significantly reduced the abundance of *Bacteroides uniformis* and *Gemmiger formicilis*, taxa previously associated with non-erosive reflux disease and esophageal carcinogenesis (51–53). These microbial shifts suggested that the clinical benefits of probiotics are mediated, at least in part, by reshaping the gut ecosystem toward a more favorable and resilient configuration.

Gut microbiota-derived metabolites are pivotal signaling molecules in the intricate host-microbe crosstalk (54). In this study, probiotic intervention led to a significant increase in succinic acid, a metabolite known to stimulate gastric acid secretion and enhance the efficacy in anti-reflux therapies when combined with PPIs (55). Notably, certain intestinal microbes can convert succinate into acetate and butyrate (56), both of which were elevated in the probiotic group. Beyond serving as the primary energy source for colonocytes, butyrate strengthens intestinal barrier function through tight junction protein modulation and exerts potent anti-inflammatory effects, offering protection in conditions such as eosinophilic esophagitis (57, 58). Additionally, probiotic supplementation modulated key neuroactive metabolites, including GABA, 5-HT, and L-tryptophan, which regulate gut motility, immune function, and visceral sensitivity (59). Dietary L-tryptophan supplementation has been reported to alleviate GERD symptoms (60). Its downstream metabolite, 5-HT, is a crucial regulator of gastrointestinal peristalsis and motility (61). However, elevated 5-HT levels may disrupt esophageal epithelial barrier function by altering tight junction protein expression, potentially contributing to GERD pathogenesis (62). Interestingly, our study also observed increased levels of L-arginine, putrescine, and spermine following probiotic intervention. L-arginine has been shown to protect against reflux esophagitis by promoting prostaglandin-mediated mucosal defense (63, 64) and may improve colonic motility (65). Its downstream metabolites, putrescine and spermine, are essential for intestinal epithelial cell proliferation, mucosal repair, and intestinal barrier function (66, 67). Together, these metabolic shifts highlight the multifaceted mechanisms by which probiotics contribute to gastrointestinal health, extending beyond microbial composition to functional metabolic reprogramming.

This study has several limitations. First, the study population was exclusively composed of Chinese individuals, which may restrict the generalizability of the findings to other ethnic and geographic populations. Second, although participants were instructed to maintain their usual dietary habits, dietary intake was not strictly controlled, potentially introducing variability in microbiota composition due to dietary influences. Furthermore, only 27 patients with esophagitis underwent subsequent endoscopy. This small sample size may lead to false-negative results in the assessment of the healing rate. Finally, although the trial was adequately powered for the primary outcome, the sample size and follow-up duration were limited. Larger, multicenter studies with extended follow-up are needed to confirm the long-term benefits and durability of probiotic effects in GERD management.

In conclusion, this randomized controlled trial demonstrates that adjunctive probiotic therapy with the Lihuo formulation is a safe and effective strategy for managing GERD, with no serious adverse events reported. It provides sustained symptom relief during and after PPI treatment, supported by targeted modulation of the gut ecosystem. The enrichment of beneficial bacteria, suppression of detrimental taxa, and increase in key bioactive metabolites like SCFAs, GABA, and succinate provide a multi-faceted mechanistic basis for the observed clinical benefits. These functional changes are consistent with the noted trend toward improved endoscopic healing, supporting the biological plausibility of our findings. These results underscore the potential of probiotics as a valuable adjunct to PPI therapy and offer a foundation for future large-scale studies powered to confirm both symptomatic and endoscopic benefits across diverse populations.

## Data Availability

All data needed to evaluate the conclusions of this study are presented in the article and the supplemental material. Raw metagenomic sequencing data have been deposited in the China National GeneBank DataBase (https://db.cngb.org/cnsa/) under accession number CNP0005091. The metabolomics data generated in this study are publicly available in the MassIVE repository under accession code MSV000095809. The bioinformatics analysis pipeline and custom scripts used for data processing are available on GitHub (https://github.com/Striverzhou/Probiotics-relieve-GERD). This study has been completed according to the STORMS Checklist (https://doi.org/10.5281/zenodo.17150958).

## SUPPLEMENTAL MATERIAL

Supplemental figures and tables msystems.01568-25-s0001.docx Fig. S1 to S5; Tables S1 to S8.

ASM does not own the copyrights to Supplemental Material that may be linked to, or accessed through, an article. The authors have granted ASM a non-exclusive, world-wide license to publish the Supplemental Material files. Please contact the corresponding author directly for reuse.
