# Supplementary material for: Adjunctive probiotic therapy sustains symptom relief in gastroesophageal reflux disease through gut microbiome-metabolome remodeling
Source: mSystems. 2026 Jan 29;11(2):e01568-25. doi: 10.1128/msystems.01568-25 (PMC12911400; doi:10.1128/msystems.01568-25)
Supplement: Supplemental figures and tables — Fig. S1 to S5; Tables S1 to S8. [file msystems.01568-25-s0001.docx]

# Table S1. Changes in Reflux Disease Questionnaire (RDQ) and Gastrointestinal Symptom Rating Scale (GSRS) Scores Over Time: Intention-to-Treat Analysis

| **Comparison and *P*-value** | | **RDQ score, mean (SD)** | **Abdominal pain**  **score, mean (SD)** | **Reflux**  **score, mean** | **Diarrhea**  **score, mean** | **Indigestion**  **score, mean** | **Constipation**  **score, mean** | **Total GSRS**  **score, mean (SD)** |
| --- | --- | --- | --- | --- | --- | --- | --- | --- |
| **Group_time point** | pla_0w (n=56) | 15.59 (7.33) | 0.65 (0.55) | 1.20 (0.76) | 0.36 (0.41) | 0.94 (0.57) | 0.36 (0.36) | 0.69 (0.32) |
|  | pla_4w (n=56) | 5.73 (5.49) | 0.32 (0.31) | 0.37 (0.57) | 0.30 (0.32) | 0.67 (0.50) | 0.37 (0.51) | 0.43 (0.26) |
|  | pla_8w (n=56) | 5.20 (5.87) | 0.31 (0.33) | 0.29 (0.47) | 0.31 (0.34) | 0.64 (0.49) | 0.37 (0.42) | 0.41 (0.28) |
|  | pla_12w (n=56) | 8.93 (7.10) | 0.42 (0.43) | 0.74 (0.72) | 0.25 (0.31) | 0.64 (0.49) | 0.31 (0.39) | 0.47 (0.28) |
|  | pro_0w (n=64) | 15.75 (7.84) | 0.58 (0.51) | 1.11 (0.64) | 0.35 (0.40) | 0.93 (0.62) | 0.31 (0.37) | 0.65 (0.35) |
|  | pro_4w (n=64) | 5.75 (6.56) | 0.36 (0.44) | 0.34 (0.47) | 0.24 (0.33) | 0.72 (0.54) | 0.17 (0.34) | 0.40 (0.27) |
|  | pro_8w (n=64) | 4.92 (5.70) | 0.31 (0.40) | 0.32 (0.39) | 0.24 (0.37) | 0.72 (0.58) | 0.21 (0.29) | 0.39 (0.31) |
|  | pro_12w (n=64) | 5.67 (4.59) | 0.33 (0.37) | 0.43 (0.41) | 0.21 (0.32) | 0.64 (0.47) | 0.15 (0.21) | 0.37 (0.24) |
| ***P*-value** | pro_0w vs pla_0w | 0.992 | 0.504 | 0.503 | 0.978 | 0.738 | 0.283 | 0.362 |
|  | pro 4w vs pla 4w | 0.758 | 0.845 | >0.999 | 0.193 | 0.558 | **0.002** | 0.42 |
|  | pro_8w vs pla_8w | 0.942 | 0.790 | 0.382 | **0.049** | 0.597 | **0.022** | 0.631 |
|  | pro_12w vs pla_12w | **0.017** | 0.255 | **0.035** | 0.423 | 0.981 | 0.054 | 0.051 |

Remarks: Statistically significant *P*-values are written in bold font. “Pla” and “pro” represent the placebo and probiotic groups, respectively. *P-*values were calculated using two-sided Wilcoxon rank-sum tests. Time points “0w”, “4w”, “8w”, and “12w” represent weeks 0, 4, 8, and 12, respectively.

# Table S2. Changes in Reflux Disease Questionnaire (RDQ) and Gastrointestinal Symptom Rating Scale (GSRS) Scores Over Time: Per-Protocol Analysis

| **Comparison and *P*-value** | | **RDQ score, mean (SD)** | **Abdominal pain score, mean (SD)** | **Reflux**  **score, mean (SD)** | **Diarrhea**  **score, mean (SD)** | **Indigestion**  **score, mean (SD)** | **Constipation**  **score, mean (SD)** | **Total GSRS score, mean (SD)** |
| --- | --- | --- | --- | --- | --- | --- | --- | --- |
| **Group_time point** | pla_0w (n=50) | 14.54 (6.41) | 0.63 (0.55) | 1.14 (0.71) | 0.36 (0.42) | 0.93 (0.60) | 0.36 (0.36) | 0.67 (0.32) |
|  | pla_4w (n=50) | 5.46 (5.41) | 0.33 (0.32) | 0.36 (0.59) | 0.30 (0.32) | 0.69 (0.51) | 0.39 (0.53) | 0.44 (0.27) |
|  | pla_8w (n=50) | 4.90 (5.94) | 0.31 (0.35) | 0.28 (0.49) | 0.32 (0.36) | 0.66 (0.51) | 0.39 (0.44) | 0.42 (0.29) |
|  | pla_12w (n=50) | 8.76 (7.31) | 0.42 (0.45) | 0.74 (0.74) | 0.25 (0.33) | 0.67 (0.51) | 0.32 (0.41) | 0.48 (0.29) |
|  | pro_0w (n=51) | 15.04 (7.71) | 0.58 (0.52) | 1.09 (0.54) | 0.37 (0.40) | 0.90 (0.63) | 0.31 (0.37) | 0.64 (0.35) |
|  | pro_4w (n=51) | 4.94 (5.91) | 0.33 (0.39) | 0.30 (0.47) | 0.21 (0.31) | 0.72 (0.54) | 0.19 (0.37) | 0.38 (0.28) |
|  | pro_8w (n=51) | 4.06 (5.21) | 0.31 (0.43) | 0.27 (0.39) | 0.23 (0.40) | 0.69 (0.55) | 0.20 (0.31) | 0.37 (0.31) |
|  | pro_12w (n=51) | 4.82 (4.56) | 0.30 (0.37) | 0.36 (0.40) | 0.22 (0.35) | 0.62 (0.47) | 0.13 (0.20) | 0.34 (0.24) |
| ***P*-value** | pro_0w vs pla_0w | 0.867 | 0.644 | 0.810 | 0.833 | 0.654 | 0.407 | 0.393 |
|  | pro_4w vs pla_4w | 0.539 | 0.646 | 0.724 | 0.108 | 0.762 | **0.005** | 0.130 |
|  | pro_8w vs pla_8w | 0.635 | 0.516 | 0.758 | **0.035** | 0.858 | **0.017** | 0.253 |
|  | pro_12w vs pla_12w | **0.007** | 0.142 | **0.010** | 0.399 | 0.647 | **0.015** | **0.012** |

Remarks: Statistically significant *P*-values are written in bold font. “Pla” and “pro” represent the placebo and probiotic groups, respectively. *P-*values were calculated using two-sided Wilcoxon rank-sum tests. Time points “0w”, “4w”, “8w”, and “12w” represent weeks 0, 4, 8, and 12, respectively.

# Table S3. Safety Evaluation of Intervention in Intention-to-Treat Population

| **Parameter** | | **Placebo group (n=56; mean± SD)** | | ***P-*value** | **Probiotic group (n=64; mean**  **±SD)** | | ***P-*value** |
| --- | --- | --- | --- | --- | --- | --- | --- |
|  |  | **Baseline period (0w)** | **Trial end point (12w)** |  | **Baseline period (0w)** | **Trial end point (12w)** |  |
| **Blood routine** | Red blood cell (10^12^/L) | 4.74±0.48 | 4.70±0.51 | 0.46 | 4.83±0.54 | 4.86±0.54 | 0.72 |
|  | White blood cell (10^9^/L) | 6.47±1.56 | 6.39±1.68 | 0.56 | 6.64±2.14 | 6.25±2.47 | 0.16 |
|  | Neutrophil (10^9^/L) | 3.75±1.30 | 3.62±1.34 | 0.43 | 4.03±1.42 | 3.58±1.00 | 0.19 |
|  | Lymphocyte (10^9^/L) | 2.14±0.55 | 2.19±0.59 | 0.69 | 2.09±1.58 | 2.15±1.96 | 0.91 |
|  | Platelet (10^9^/L) | 229.93±47.59 | 223.93±45.27 | 0.49 | 229.11±66.89 | 214.82±66.21 | 0.18 |
|  | Hemoglobin (g/L) | 143.79±15.26 | 141.07±16.27 | 0.41 | 144.88±14.65 | 144.21±17.52 | 0.98 |
|  | Hematocrit (L/L) | 0.43±0.04 | 0.43±0.05 | 0.45 | 0.44±0.04 | 0.44±0.05 | 0.95 |
| **Serum biochemistry** | Alanine aminotransferase (U/L) | 24.40±10.95 | 23.70±12.03 | 0.61 | 30.29±21.10 | 26.46±17.91 | 0.32 |
|  | Aspartate aminotransferase (U/L) | 24.10±5.86 | 24.20±5.90 | 0.98 | 25.67±12.48 | 24.40±8.43 | 0.96 |
|  | Total bilirubin (μmol/L) | 16.37±6.29 | 15.36±5.73 | 0.36 | 17.93±8.04 | 16.65±4.85 | 0.94 |
|  | Creatinine (μmol/L) | 76.15±14.53 | 75.21±12.66 | 0.75 | 74.13±15.82 | 73.30±14.64 | 0.88 |
|  | Blood urea nitrogen (mmol/L) | 4.78±1.15 | 4.57±0.91 | 0.37 | 4.55±1.21 | 4.56±1.04 | 0.94 |
| **Urine routine** | Urine red cell | Normal | Normal | - | Normal | Normal | - |
|  | Urine white cell | Normal | Normal | - | Normal | Normal | - |
|  | Urine protein | Normal | Normal | - | Normal | Normal | - |
|  | Ketone | Normal | Normal | - | Normal | Normal | - |
| **Stool routine** | Occult blood | Normal | Normal | - | Normal | Normal | - |
| **Electrocardiogram (ECG)** | **--** | Normal | Normal | - | Normal | Normal | - |

Remarks: *P*-values were calculated using Chi-square tests or two-sided Wilcoxon rank-sum tests. Time points “0w” and “12w” represent weeks 0 and 12, respectively.

| **Table S4. Adverse Events Reported in Intention-to-Treat Population** | | | |
| --- | --- | --- | --- |
| **Adverse Events** | **Placebo group**  **(n=56)** | **Probiotic group**  **(n=64)** | ***P-*value** |
| **Total reported non-severe adverse events** | 8 (14.29%) | 17 (26.56%) | 0.10 |
| Upper respiratory tract infection | 2 (3.57%) | 6 (9.38%) | 0.37 |
| Gingivitis | 1 (1.79%) | 2 (3.12%) | > 0.99 |
| Diarrhea | 1 (1.79%) | 1 (1.56%) | > 0.99 |
| Others | 4 (7.14%) | 8 (17.19%) | 0.50 |
| **Reported severe adverse events** | 1 (1.79%) | 0 (0%) | 0.47 |
| Remark: *P*-values were calculated using Chi-square tests or Fisher’s exact tests. | | | |

# Table S5. Significantly Differential Species-Level Genome Bins (SGBs) Between Probiotic and Placebo Groups During and After the Intervention

| **Time** | **SGB ID** | **Species-level taxonomy** | **Significantly differential SGBs in probiotic (pro) and placebo (pla) groups at weeks 0, 4, 8, and 12** | | | | ***P*-value, two-sided Wilcoxon rank-sum test** | |
| --- | --- | --- | --- | --- | --- | --- | --- | --- |
|  |  |  | **pro_0w (mean±SD)** | **pla_0w (mean±SD)** | **pro (mean±SD)** | **pla (mean±SD)** | **pro_0w vs pla_0w** | **pro vs pla** |
| **Week_4** | **SGB.101** | *Fimenecus* sp000432435 | 0.03±0.08 | 0.121±0.428 | 0.126±0.361 | 0.398±1.015 | 0.983 | **0.050** |
|  | **SGB.102** | *Phocaeicola plebeius* | 0.571±2.572 | 0.903±2.038 | 0.324±1.717 | 0.628±1.588 | 0.188 | **0.041** |
|  | **SGB.119** | *Erysipelatoclostridium ramosum* | 0.037±0.084 | 0.041±0.106 | 0.013±0.027 | 0.006±0.038 | 0.833 | **0.004** |
|  | **SGB.140** | *Clostridium_*P *perfringens* | 0.023±0.134 | 0.046±0.203 | 0.032±0.071 | 0.013±0.048 | 0.691 | **0.029** |
|  | **SGB.162** | *Coprobacter fastidiosus* | 0.004±0.021 | 0.008±0.023 | 0.003±0.023 | 0.038±0.1 | 0.287 | **0.004** |
|  | **SGB.191** | *Ruminococcus_*E sp003526955 | 0.196±1.029 | 0.184±0.686 | 0.196±0.793 | 0.677±1.773 | 0.238 | **0.015** |
|  | **SGB.192** | *KLE1615* sp900066985 | 0.049±0.09 | 0.054±0.115 | 0.117±0.192 | 0.171±0.213 | 0.975 | **0.041** |
|  | **SGB.261** | *Butyricimonas faecihominis* | 0.018±0.075 | 0.02±0.052 | 0.011±0.041 | 0.031±0.065 | 0.253 | **0.045** |
|  | **SGB.269** | *Bifidobacterium animalis* | 0±0 | 0±0 | 0.176±0.301 | 0±0 | >0.999 | **0.000** |
|  | **SGB.325** | *Lactiplantibacillus plantarum* | 0±0 | 0±0 | 0.051±0.096 | 0±0 | >0.999 | **0.000** |
|  | **SGB.326** | *Faecalimonas umbilicata* | 0.07±0.149 | 0.037±0.087 | 0.116±0.377 | 0.016±0.046 | 0.438 | **0.037** |
|  | **SGB.389** | *Bacteroides intestinalis_*A | 0.065±0.317 | 0.03±0.169 | 0.072±0.311 | 0.147±0.384 | 0.681 | **0.048** |
|  | **SGB.40** | *Bacteroides uniformis* | 2.069±3.202 | 1.62±2.049 | 1.706±3.012 | 2.226±2.407 | 0.669 | **0.029** |
|  | **SGB.405** | *Haemophilus_*D *parainfluenzae_*K | 0.006±0.032 | 0.006±0.033 | 0.118±0.19 | 0.049±0.128 | >0.999 | **0.017** |
|  | **SGB.42** | *Acetatifactor intestinalis* | 0.136±0.376 | 0.162±0.398 | 0.233±0.606 | 0.461±0.712 | 0.510 | **0.007** |
|  | **SGB.46** | *Alistipes onderdonkii* | 0.486±2.103 | 0.194±0.395 | 0.272±0.699 | 0.266±0.291 | 0.351 | **0.015** |
|  | **SGB.47** | *Agathobacter rectalis* | 0.909±1.676 | 0.785±1.27 | 1.354±2.059 | 1.71±1.824 | 0.346 | **0.045** |
|  | **SGB.49** | *Alistipes putredinis* | 0.608±1.279 | 0.738±1.13 | 0.75±1.524 | 1.194±1.177 | 0.169 | **0.005** |
|  | **SGB.63** | *Dysosmobacter* sp001916835 | 0.075±0.111 | 0.092±0.171 | 0.105±0.163 | 0.195±0.231 | 0.818 | **0.042** |
|  | **SGB.64** | *Butyricimonas virosa* | 0.032±0.078 | 0.034±0.08 | 0.051±0.112 | 0.075±0.189 | 0.693 | **0.027** |
|  | **SGB.68** | *Phocaeicola massiliensis* | 0.32±0.783 | 1.197±2.61 | 0.363±0.881 | 1.632±2.922 | 0.086 | **0.012** |
|  | **SGB.7** | *Bacteroides fragilis* | 2.318±4.953 | 4.141±12.738 | 1.091±2.64 | 1.062±4.808 | 0.604 | **0.042** |
|  | **SGB.81** | *Gemmiger formicilis* | 0.087±0.226 | 0.159±0.338 | 0.112±0.215 | 0.195±0.304 | 0.262 | **0.026** |
|  | **SGB.91** | *Odoribacter splanchnicus* | 0.149±0.211 | 0.124±0.187 | 0.154±0.276 | 0.258±0.329 | 0.670 | **0.035** |
|  | **SGB.93** | *Bacteroides clarus* | 0.047±0.251 | 0.09±0.393 | 0.016±0.077 | 0.208±1.085 | 0.199 | **0.014** |
|  | **SGB.106** | *Lachnospira eligens_*A | 0.048±0.147 | 0.1±0.389 | 0.013±0.051 | 0.135±0.329 | 0.826 | **0.023** |
|  | **SGB.139** | *Copromonas* sp900066785 | 0.038±0.071 | 0.083±0.132 | 0.071±0.296 | 0.096±0.156 | 0.087 | **0.039** |
|  | **SGB.154** | *Faecalibacterium prausnitzii* J | 0.144±0.42 | 0.128±0.272 | 0.057±0.187 | 0.303±0.594 | 0.547 | **0.014** |
|  | **SGB.172** | *Bifidobacterium longum* | 0.1±0.275 | 0.212±0.539 | 0.383±1.411 | 0.064±0.236 | 0.329 | **0.014** |
|  | **SGB.18** | *Allisonella histaminiformans* | 0.015±0.055 | 0.013±0.033 | 0.026±0.053 | 0.004±0.014 | 0.958 | **0.017** |
|  | **SGB.225** | *Choladocola* sp003480725 | 0.065±0.094 | 0.077±0.094 | 0.047±0.079 | 0.075±0.099 | 0.366 | **0.044** |

| **Week_8** | **SGB.234** | *Alistipes* A *ihumii* | 0.002±0.01 | 0.015±0.072 | 0.009±0.054 | 0.012±0.031 | 0.428 | **0.032** |
| --- | --- | --- | --- | --- | --- | --- | --- | --- |
|  | **SGB.269** | *Bifidobacterium animalis* | 0±0 | 0±0 | 0.357±0.556 | 0.005±0.028 | >0.999 | **0.000** |
|  | **SGB.272** | *Blautia_*A sp003471165 | 0.238±0.488 | 0.115±0.2 | 0.32±0.387 | 0.144±0.168 | 0.090 | **0.021** |
|  | **SGB.325** | *Lactiplantibacillus plantarum* | 0±0 | 0±0 | 0.114±0.243 | 0±0 | >0.999 | **0.000** |
|  | **SGB.326** | *Faecalimonas umbilicata* | 0.07±0.149 | 0.037±0.087 | 0.078±0.145 | 0.016±0.061 | 0.438 | **0.002** |
|  | **SGB.4** | *Enterocloster bolteae* | 0.297±0.704 | 0.183±0.555 | 0.161±0.316 | 0.03±0.059 | 0.249 | **0.009** |
|  | **SGB.40** | *Bacteroides uniformis* | 2.069±3.202 | 1.62±2.049 | 1.114±1.779 | 2.469±3.312 | 0.669 | **0.019** |
|  | **SGB.42** | *Acetatifactor intestinalis* | 0.136±0.376 | 0.162±0.398 | 0.285±0.861 | 0.523±1.059 | 0.510 | **0.028** |
|  | **SGB.46** | *Alistipes onderdonkii* | 0.486±2.103 | 0.194±0.395 | 0.226±0.527 | 0.372±0.677 | 0.351 | **0.050** |
|  | **SGB.49** | *Alistipes putredinis* | 0.608±1.279 | 0.738±1.13 | 0.37±0.665 | 1.057±1.131 | 0.169 | **0.001** |
|  | **SGB.51** | *Bacteroides caccae* | 0.504±0.917 | 0.482±0.756 | 0.308±0.586 | 0.46±0.557 | 0.624 | **0.025** |
|  | **SGB.60** | *Ventrimonas* sp003480315 | 0.03±0.048 | 0.074±0.153 | 0.028±0.043 | 0.069±0.081 | 0.055 | **0.011** |
|  | **SGB.63** | *Dysosmobacter* sp001916835 | 0.075±0.111 | 0.092±0.171 | 0.079±0.142 | 0.161±0.202 | 0.818 | **0.045** |
|  | **SGB.68** | *Phocaeicola massiliensis* | 0.32±0.783 | 1.197±2.61 | 0.26±0.889 | 1.196±2.059 | 0.086 | **0.002** |
|  | **SGB.7** | *Bacteroides fragilis* | 2.318±4.953 | 4.141±12.738 | 1.444±4.016 | 0.227±0.522 | 0.604 | **0.046** |
|  | **SGB.81** | *Gemmiger formicilis* | 0.087±0.226 | 0.159±0.338 | 0.079±0.158 | 0.226±0.338 | 0.262 | **0.011** |
|  | **SGB.90** | *Eisenbergiella* sp900066775 | 0.036±0.055 | 0.063±0.125 | 0.045±0.086 | 0.102±0.142 | 0.490 | **0.016** |
| **Week_12** | **SGB.152** | *Wujia chipingensis* | 0.092±0.29 | 0.127±0.476 | 0.386±1.003 | 0.205±0.687 | 0.647 | **0.036** |
|  | **SGB.154** | *Faecalibacterium prausnitzii*_J | 0.144±0.42 | 0.128±0.272 | 0.053±0.164 | 0.274±0.65 | 0.547 | **0.021** |
|  | **SGB.155** | *Lachnospira* sp000437735 | 0.297±0.784 | 0.089±0.233 | 0.817±1.654 | 0.209±0.474 | 0.061 | **0.033** |
|  | **SGB.162** | *Coprobacter fastidiosus* | 0.004±0.021 | 0.008±0.023 | 0.003±0.02 | 0.06±0.189 | 0.287 | **0.029** |
|  | **SGB.225** | *Choladocola* sp003480725 | 0.065±0.094 | 0.077±0.094 | 0.037±0.047 | 0.073±0.079 | 0.366 | **0.041** |
|  | **SGB.269** | *Bifidobacterium animalis* | 0±0 | 0±0 | 0.106±0.293 | 0±0 | >0.999 | **0.000** |
|  | **SGB.325** | *Lactiplantibacillus plantarum* | 0±0 | 0±0 | 0.023±0.06 | 0±0 | >0.999 | **0.005** |
|  | **SGB.33** | *Clostridium_*Q *fessum* | 0.234±0.303 | 0.239±0.249 | 0.173±0.209 | 0.289±0.323 | 0.434 | **0.049** |
|  | **SGB.392** | *Clostridium* sp900540255 | 0.017±0.045 | 0.011±0.028 | 0.491±1.467 | 0.021±0.075 | 0.596 | **0.014** |
|  | **SGB.40** | *Bacteroides uniformis* | 2.069±3.202 | 1.62±2.049 | 1.228±2.165 | 2.107±2.428 | 0.669 | **0.028** |
|  | **SGB.60** | *Ventrimonas* sp003480315 | 0.03±0.048 | 0.074±0.153 | 0.049±0.143 | 0.068±0.074 | 0.055 | **0.014** |
|  | **SGB.80** | *Faecalibacterium duncaniae* | 0.422±0.916 | 0.775±1.245 | 0.462±0.84 | 1.009±1.258 | 0.089 | **0.049** |

Remarks: Statistically significant *P-*values are written in bold font. Time points “0w”, “4w”, “8w”, and “12w” represent weeks 0, 4, 8, and 12, respectively.

# Table S6. Significantly Differential Predicted Bioactive Gut Metabolites Between Probiotic and Placebo Groups During and After the Intervention

| **Predicted metabolite** | | **X2.hydroxyp-**  **henethylamine** | **chenodeoxycholate** | **X7.methylguanine** | **phytosphingosine** | **imidazole.**  **propionate** | **citrulline** | **N.acetylpu**  **trescine** | **glutamate** |
| --- | --- | --- | --- | --- | --- | --- | --- | --- | --- |
| **Significantly differential bioactive gut metabolites in the probiotic (pro) and placebo (pla) groups at weeks 0, 4, 8, and 12** | **Mean pro 0w** | 1.18E-04 | 3.89E-04 | 3.11E-04 | 6.55E-05 | 4.21E-04 | 4.20E-04 | 2.55E-04 | 3.75E-03 |
|  | **Mean pro 4w** | 1.33E-04 | 4.25E-04 | 3.01E-04 | 7.40E-05 | 4.86E-04 | 4.01E-04 | 2.81E-04 | 3.88E-03 |
|  | **Mean pro_8w** | 1.36E-04 | 4.54E-04 | 3.03E-04 | 7.63E-05 | 5.35E-04 | 4.47E-04 | 3.06E-04 | 4.10E-03 |
|  | **Mean pro_12 w** | 1.05E-04 | 3.60E-04 | 2.98E-04 | 6.58E-05 | 5.01E-04 | 4.48E-04 | 2.27E-04 | 3.82E-03 |
|  | **Mean_pla_0w** | 1.03E-04 | 3.90E-04 | 3.08E-04 | 6.64E-05 | 3.69E-04 | 4.15E-04 | 2.54E-04 | 3.55E-03 |
|  | **Mean pla 4w** | 9.80E-05 | 3.24E-04 | 2.87E-04 | 6.59E-05 | 4.12E-04 | 3.34E-04 | 2.16E-04 | 3.80E-03 |
|  | **Mean pla 8w** | 1.24E-04 | 4.10E-04 | 2.97E-04 | 7.06E-05 | 3.82E-04 | 3.53E-04 | 2.27E-04 | 3.77E-03 |
|  | **Mean_pla_12 w** | 1.08E-04 | 2.86E-04 | 2.87E-04 | 6.29E-05 | 4.01E-04 | 3.81E-04 | 2.35E-04 | 3.82E-03 |
|  | **SD_pro_0w** | 4.62E-05 | 1.95E-04 | 3.00E-05 | 1.48E-05 | 2.33E-04 | 2.12E-04 | 1.30E-04 | 5.76E-04 |
|  | **SD pro 4w** | 6.59E-05 | 2.05E-04 | 3.77E-05 | 1.79E-05 | 2.53E-04 | 2.39E-04 | 1.58E-04 | 6.51E-04 |
|  | **SD pro 8w** | 7.13E-05 | 1.94E-04 | 3.77E-05 | 1.82E-05 | 2.68E-04 | 2.16E-04 | 1.75E-04 | 6.36E-04 |
|  | **SD pro 12w** | 4.05E-05 | 1.97E-04 | 3.57E-05 | 1.86E-05 | 2.69E-04 | 2.12E-04 | 1.31E-04 | 6.61E-04 |
|  | **SD pla 0w** | 3.58E-05 | 1.88E-04 | 3.21E-05 | 1.74E-05 | 1.83E-04 | 2.35E-04 | 1.37E-04 | 4.41E-04 |
|  | **SD pla 4w** | 5.84E-05 | 1.72E-04 | 3.58E-05 | 1.30E-05 | 1.79E-04 | 1.98E-04 | 1.19E-04 | 5.58E-04 |
|  | **SD pla 8w** | 8.38E-05 | 2.76E-04 | 3.51E-05 | 1.79E-05 | 1.88E-04 | 2.35E-04 | 1.33E-04 | 4.67E-04 |
|  | **SD pla 12w** | 6.78E-05 | 1.65E-04 | 3.01E-05 | 1.80E-05 | 1.94E-04 | 2.63E-04 | 1.68E-04 | 4.76E-04 |
| ***P-*value, two- sided Wilcoxon rank-sum test** | **pro 0w vs pla 0w** | 0.185 | 0.876 | 0.724 | 0.882 | 0.335 | 0.837 | 0.972 | 0.095 |
|  | **pro 4w vs pla 4w** | **0.007** | **0.019** | **0.027** | **0.046** | 0.215 | 0.140 | 0.052 | 0.560 |
|  | **pro 8w vs pla 8w** | 0.268 | 0.123 | 0.285 | 0.146 | **0.004** | **0.020** | **0.038** | **0.044** |
|  | **pro 12w vs pla 12w** | 0.557 | 0.097 | 0.167 | 0.371 | 0.178 | 0.095 | 0.713 | 0.565 |

Remarks: Statistically significant *P-*values are written in bold font. Time points “0w”, “4w”, “8w”, and “12w” represent weeks 0, 4, 8, and 12, respectively.

**Table S7. Significantly Differential Fecal Metabolites Between Probiotic and Placebo Groups During and After the Intervention**


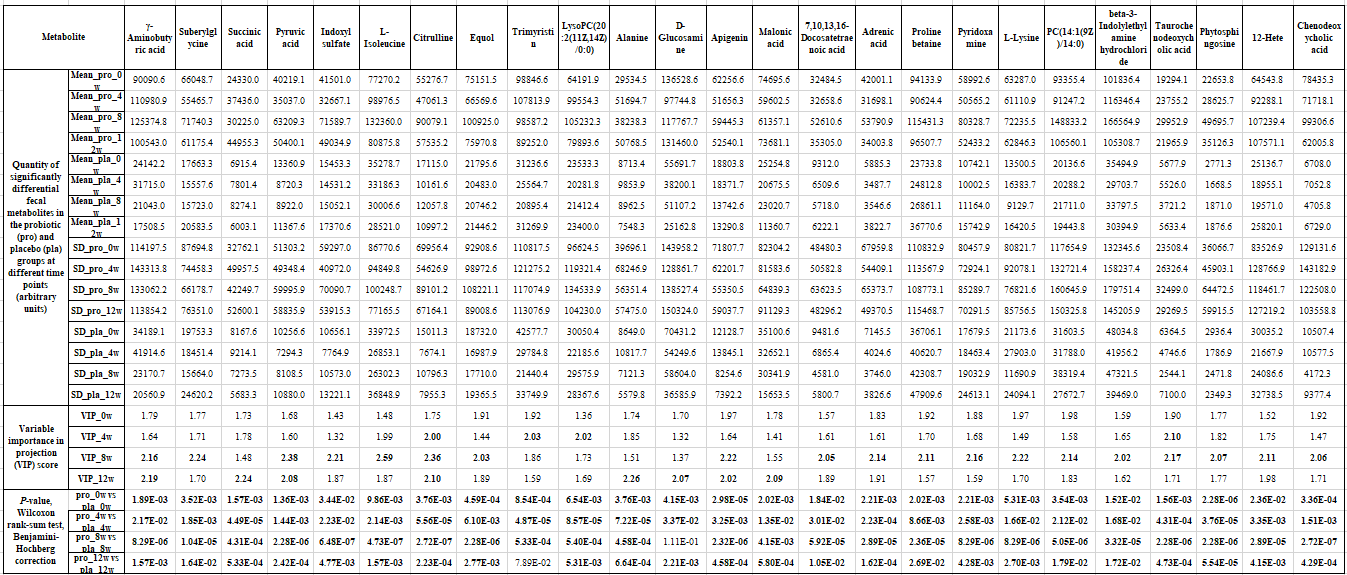


Remarks: Statistically significant *P*-values are written in bold font. Time points “0w”, “4w”, “8w”, and “12w” represent weeks 0, 4, 8, and 12, respectively.

**Table S8. Significantly Differential** **Fecal Neuroactive Metabolites and Short-chain Fatty Acids** **Between Probiotic and Placebo Groups During and After the Intervention**

Remarks: Statistically significant *P*-values are written in bold font. Time points “0w”, “4w”, “8w”, and “12w” represent weeks 0, 4, 8, and 12, respectively.


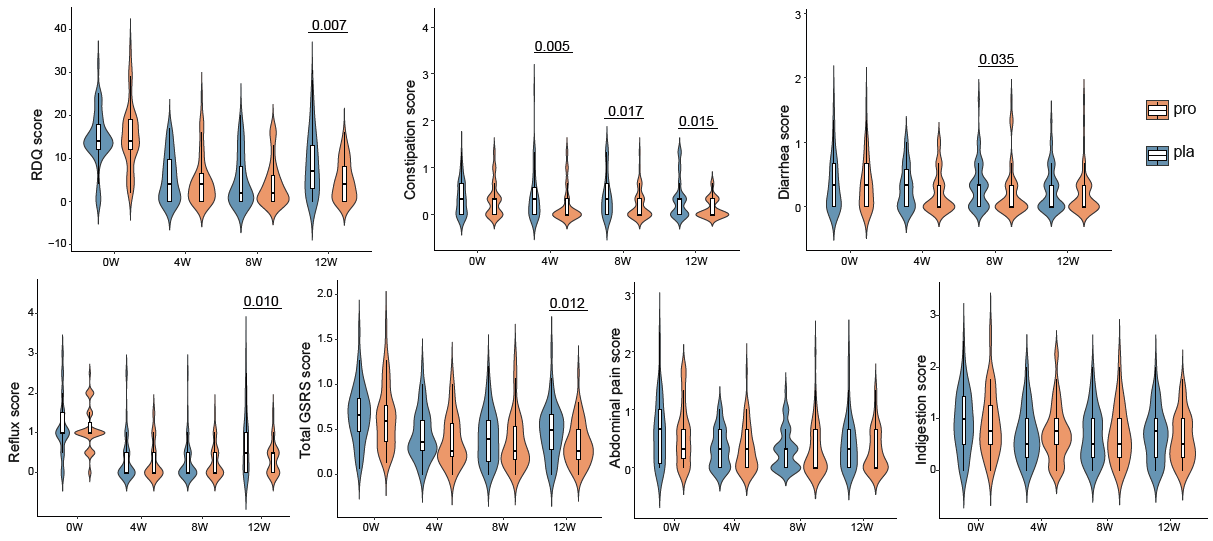


**Figure S1. Probiotic supplementation sustains reflux relief after PPI cessatio in the per-protocol population.** Changes in Reflux Disease Questionnaire (RDQ) score and Gastrointestinal Symptom Rating Scale (GSRS) total and subscores (constipation, diarrhea, reflux, abdominal pain, indigestion) over 12 weeks in the per-protocol population. Data are shown for the probiotic (pro; n = 51) and placebo (pla; n = 50) groups at weeks 0 (0w), 4 (4w), 8 (8w), and 12 (12w). Intergroup differences at each time point were assessed using two-side Wilcoxon rank-sum tests, with significant *P*-values (*P* < 0.05) indicated. In the violin plots, the box represents the interquartile range, with the median indicated by the internal line. Whiskers extend to the lowest and highest values within 1.5 times the interquartile range.


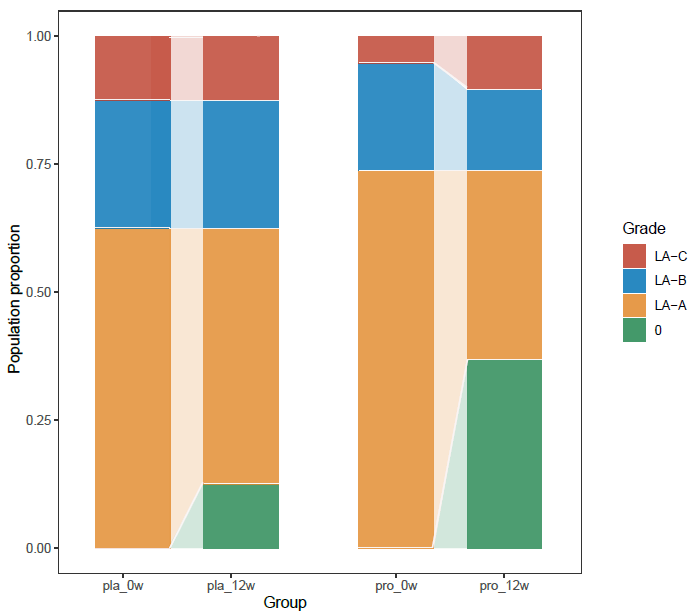


**Figure S2. Probiotic intervention is associated with greater endoscopic healing in GERD patients.** Distribution of esophagitis severity according to the Los Angeles (LA) classification in the probiotic (pro; n = 19) and placebo (pla; n = 8) groups at week 0 (0w) and after 12 weeks (12w) of intervention. Stacked bar charts depict the proportion of patients with LA grades A, B, C, or healed mucosa (grade 0).


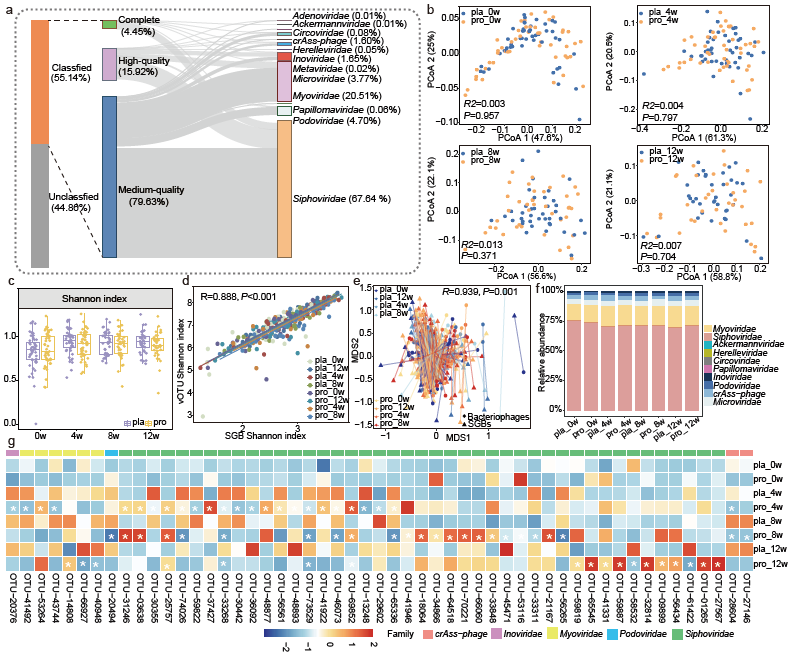


**Figure S3. High degree of congruence in compositional variation between bacterial and bacteriophage communities.** (a) Completeness, quality classification, and family-level taxonomic distribution of annotated viral operational taxonomic units (vOTUs) in the probiotic (pro; n = 49) and placebo (pla; n = 49) groups. (b) Principal coordinates analysis (Bray-Curtis dissimilarity) score plots of the gut phageome of the two groups at weeks 0 (0w), 4 (4w), 8 (8w), and 12 (12w). (c) Shannon diversity index of the gut phageome in both groups across time points. (d) Shannon index correlation analysis and (e) Procrustes analysis confirm high concordance between bacterial (species-level genome bin, SGB-based) and bacteriophage (vOTU) community structures along the study. (f, g) Phageome composition: (f) family-level distribution and (g) significant differentially abundant vOTUs at specific time points. Statistical significance was determined using two-sided Wilcoxon rank-sum tests and is indicated with an asterisk (* *P* < 0.05). The color scale represents relative abundance, ranging from high (red) to low (blue).


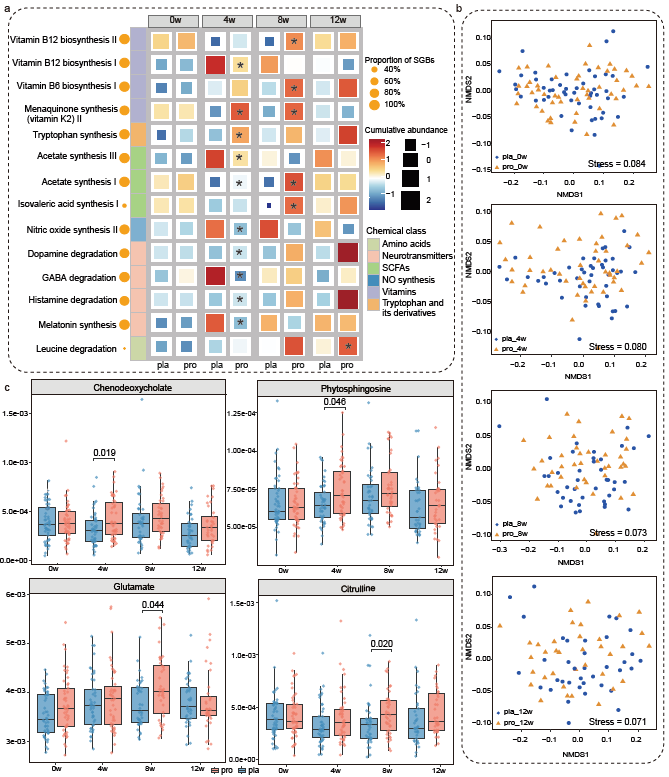


**Figure S4. Probiotic intervention reshapes microbial metabolic potential and predicted metabolite profiles.** (a) Heatmap showing cumulative abundance of 14 significantly different gut metabolic modules (GMMs), between the probiotic (pro; n = 49) and placebo (pla; n = 49) groups at weeks 0 (0w), 4 (4w), 8 (8w), and 12 (12w). Circle size (on the left of the heatmap) reflects the proportion of species-level genome bins (SGBs) encoding each module. Intergroup differences were evaluated using two-sided Wilcoxon rank-sum tests (* *P* < 0.05). The color scale (red to blue) and square size reflect the cumulative abundance of each feature, with darker red and larger squares indicating higher abundance, and blue and smaller squares indicating lower abundance. (b) Non-metric multidimensional scaling of GMM profiles of the two groups at the indicated time points. (c) Boxplots of differential bioactive metabolites that were responsive to the adjunctive probiotic treatment, derived from MelonnPan analysis. Significant intergroup differences within the same time point are indicated, evaluated using two-sided Wilcoxon rank-sum tests (*P* < 0.05). Boxes represent interquartile range with median line; whiskers extend to 1.5×interquartile range. SCFAs, short-chain fatty acids; NO, nitric oxide.


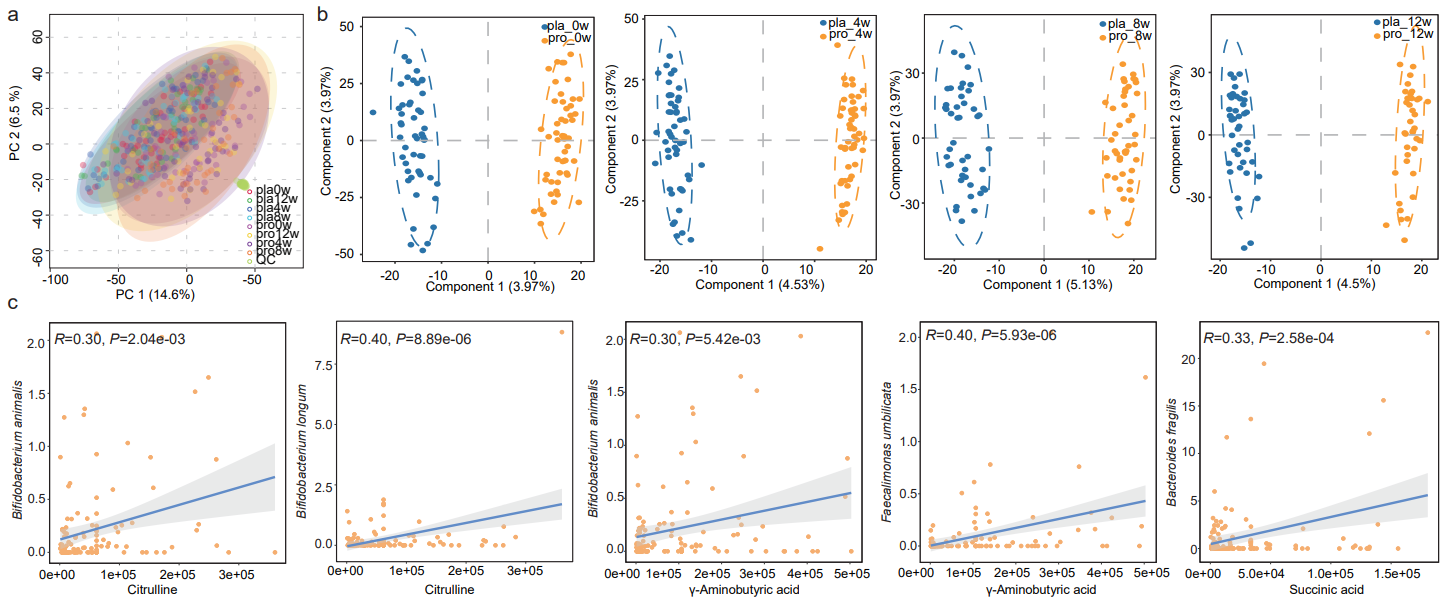


**Figure S5.** **Probiotic supplementation induces distinct fecal metabolomic shifts and microbe-metabolite correlations.** (a) Principal component analysis and (b) orthogonal partial least squares-discriminant analysis score plots of the fecal metabolomes from the probiotic (pro; n = 51) and placebo (pla; n = 50) groups at weeks 0 (0w), 4 (4w), 8 (8w), and 12 (12w). Quality control (QC) samples are indicated. (c) Pearson correlation scatter plots illustrating significant associations between differentially abundant fecal metabolites and bacterial species (derived from species-level genome bins) in the probiotic group. The blue line represents the linear regression fit; the gray band indicates the 95% confidence interval.
